# Building an extensible cell wall

**DOI:** 10.1093/plphys/kiac184

**Published:** 2022-04-23

**Authors:** Daniel J Cosgrove

**Affiliations:** Department of Biology, Penn State University, Pennsylvania 16802, USA

## Abstract

This article recounts, from my perspective of four decades in this field, evolving paradigms of primary cell wall structure and the mechanism of surface enlargement of growing cell walls. Updates of the structures, physical interactions, and roles of cellulose, xyloglucan, and pectins are presented. This leads to an example of how a conceptual depiction of wall structure can be translated into an explicit quantitative model based on molecular dynamics methods. Comparison of the model’s mechanical behavior with experimental results provides insights into the molecular basis of complex mechanical behaviors of primary cell wall and uncovers the dominant role of cellulose–cellulose interactions in forming a strong yet extensible network.

## Teaser

The ordered synthesis and bundling of cellulose microfibrils leads to a strong yet extensible surface network whose organization and physical properties are modulated by pliant matrix polysaccharides.

## Introduction

Plant growth and morphogenesis depend on the remarkable ability of plant cell walls to extend irreversibly, often increasing in surface area >100-fold before growth ceases. Such growth requires an extensible wall, adequate turgor pressure, and wall loosening to enable the wall to yield to the tensile forces generated by cell turgor. The discovery in my lab of wall loosening by α-expansin (McQueen-Mason et al., 1992) proved surprising because its biophysical actions seemed incompatible with prevailing concepts of cell wall architecture. When applied to isolated cell walls clamped in tension, α-expansin induced irreversible extension within seconds, without evidence of enzymatic action. Wall extension could continue for hours in acidic buffer without evidence for mechanical softening of the wall, changes in pectin stiffness, or cutting of hypothetical xyloglucan tethers between cellulose microfibrils (CMFs). Moreover, wall extension could continue for hours in acidic buffer and be turned on or off rapidly and repeatedly by swapping acidic and neutral buffers. No other wall protein, enzymatic or not, has been shown to exert a similar effect. What arrangement of cell wall polymers is consistent with these actions?

The backstory to this review can be traced to my graduate years in Paul Green’s lab at Stanford University in the late 1970s; as part of my PhD research, I investigated the rapid suppression of hypocotyl elongation by blue light: ∼20 s after onset of light, the growth rate (GR) declined precipitously, with a halftime of ∼15 s (Cosgrove, 1981b). A plausible mechanism was that turgor decreased rapidly in response to blue light, but that proved not to be the case (Cosgrove and Green, 1981; Cosgrove, 1988). Instead, cell wall stress relaxation and yielding were inhibited, yet with little effect on the mechanical extensibility of isolated walls. A likely explanation was worked out later by my first PhD student, Edgar Spalding, who discovered that blue light induced a massive electrical depolarization of the plasma membrane brought on by inactivation of the plasma membrane H^+^-ATPase (Spalding and Cosgrove, 1992). Presumably the ensuing increase in wall pH reduced the activity of α-expansins in the wall, but those inferences were later tested only partially in a different plant system (Okazaki et al., 1995). My research diverged from photobiology at this point as our discovery of expansins, led by my second PhD student, Simon McQueen-Mason, took center stage (McQueen-Mason et al., 1992). It became clear that to understand expansin’s puzzling activity required a synthesis of cell wall structure with polymer mechanics and protein-mediated creep. This Founders’ Review is my attempt to fill in this part of the story, although it is still incomplete.

This article focuses on the molecular architecture of the growing cell wall and the physical basis of its assembly and extensibility. The emerging view is of a supramolecular polymer complex based on noncovalent binding interactions. With indirect guidance from within the cell, three functionally distinct polysaccharides assemble at the cell surface to make a strong anisotropic network, often multilayered. I make the case that the physical network of CMFs harnesses and channels the mechanical forces generated by cell turgor to power sliding of CMFs within the elastically stretched network. Such sliding may induce wall stress relaxation and thereby cellular water uptake and cell enlargement. There is growing evidence of cell wall surveillance systems that modify the wall by processes involving surface receptors, the cytoskeleton and membrane transporters, affecting the wall in complex ways. These provide the potential for complex feedback loops in living cells to stabilize the growing wall and to reprogram it dynamically to adjust GRs and expansion patterns.

Two decades ago, I published in this journal a brief update of primary cell wall structure and mechanisms of wall loosening (Cosgrove, 2001). At that time, there were varied depictions of growing cell wall architecture, most implying a central role for xyloglucan in cell wall structure and mechanics. In the intervening years, much has changed as the biochemical data that formed the foundation for early molecular concepts of wall structure were augmented with results from genetics and cell biology as well as various spectroscopic approaches, atomic force microscopy (AFM), mechanical analyses, computational modeling, and more. These more recent results have highlighted the complex properties of the cell wall and the molecular processes underpinning its assembly and growth, but they have also undermined long-accepted concepts of wall architecture and widened the disparity of views among current researchers about the nature of cell wall growth. Today we encounter diverse views concerning the structural and mechanical roles of cellulose, xyloglucan, and pectin, to be reviewed below. While I focus on conclusions and results that seem most compelling to me, I also attempt to point out other views so the reader is alerted to alternative ideas and where additional research is needed. This review is mostly about diffusely growing cell walls and deals only tangentially with tip growth where intense local surface enlargement is closely coupled to deposition of wall materials. Although the two growth patterns are often treated separately, they likely share some common features of wall extension (Dumais, 2021).

## What is an extensible cell wall?

Lockhart (1965) defined wall extensibility (φ) as a coefficient relating steady GR to turgor pressure (P) above a yield threshold (Y): GR = φ(P−Y). This equation provides the simplest explicit definition of wall extensibility in the context of cell growth and has been widely adopted (Smithers et al., 2019; Dumais, 2021), but it has its critics (Chakraborty et al., 2021). It was formulated as an empirical equation that was not based on cell wall structure or the physical interactions of wall polymers. Later, my colleague Boris Veytsman, a polymer physicist, derived Lockhart’s equation from first principles of the thermodynamics of cell wall stretching, concluding that this relation is valid for any realistic cell wall structure (Veytsman and Cosgrove, 1998). We also showed how this behavior may arise from the thermodynamics of a polymer-based material with reversible binding between polymers. Thus Lockhart’s equation is a very generic one applicable to a variety of cell wall structures. However, I now consider the predominant concept of wall structure at that time, in which stiff CMFs are linked only through xyloglucan tethers, to be unlikely, for reasons discussed below.

Experimentally, high-resolution tracking of GRs after abrupt steps in turgor showed that φ and Y can change dynamically, and hence they are not static properties of the wall (Green and Cummins, 1974; Okamoto et al., 1990). How these adjustments occur mechanistically remains unclear, in part because this biophysical formulation is silent regarding the molecular nature of cell wall enlargement. Dumais (2021) recast Lockhart’s ideas in the form of differential equations and proposed ideas about how chemical and mechanical catalysis might be added to this mathematical formalism. This could be a very useful refinement if connected to wall structure and changing polymer interactions during cell wall extension.

Cell wall growth usually entails slow, irreversible separation and/or sliding of CMFs and other wall polymers at constant wall stress, generated by cell turgor. It is thought to be a kind of polymer creep. For this review, cell walls with the ability to grow (increase in surface area irreversibly) are considered extensible, without prejudice to the exact mechanism of irreversible surface area enlargement. It has long been recognized that wall extensibility in this growth sense differs from mechanical properties measured when a cell wall is rapidly stretched by an applied force, for example, see remarks by Cleland (1967) and by Taiz (1984). These two wall extensions differ because they operate at different time scales and because the motions have different drivers; in the latter case, it is driven by an increase in force carried by the wall network, while in the first case it results from a lowering of the energy barrier limiting polymer motion at constant force. This distinction is often obscured in contemporary studies that assume they are equivalent.

How do different measures of wall mechanics relate to one another? To answer this question we compared wall mechanics of onion (*Allium cepa*) epidermal strips as assessed by tensile stretching, tensile creep, surface indentation, and lateral mobility of CMFs (Zhang et al., 2019) (see Box 1). We used different enzymes to probe the contribution of specific wall polysaccharides to these biomechanical measures. Each technique reported a distinctive set of responses, showing that one cannot reliably translate results from one method to the other. For example, treatment with pectate lyase greatly reduced the resistance of the wall to surface indentation and increased the lateral mobility of CMFs, but had negligible effect on tensile stiffness or on tensile creep. The results demonstrate the importance of pectins for indentation mechanics, but challenge a common assumption that surface indentation measures wall extensibility in the plane of the wall.

**Figure:**
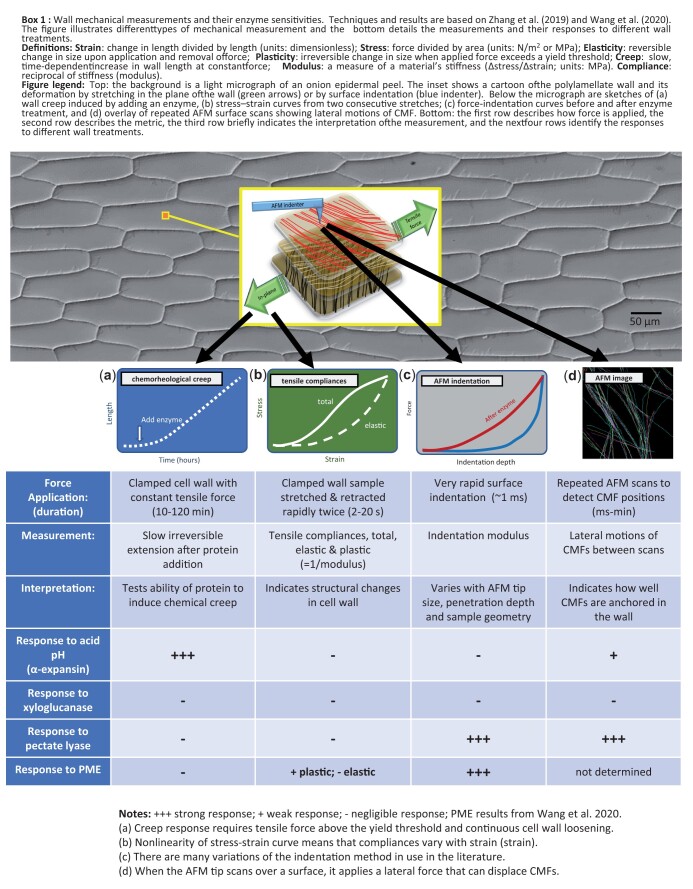


The distinctive responses of wall elasticity, plasticity, and creep (see definitions in Box 1) indicate different polymer motions underlie these mechanical properties. This conclusion is relevant to attempts to relate wall elasticity to irreversible deformations (plasticity, creep, and growth). Zhang et al. (2019) suggested the terms “softening” for processes that make the wall more deformable to applied force and “loosening” for processes that increase cell wall creep at constant force. Loosening and softening are not tightly linked. One take-home message: wall mechanics is a multifaceted concept where the details of force application (its timing, magnitude, and direction relative to structural anisotropies of the wall) matter a great deal for the results and their interpretation.

Spatial patterns of growth, and presumably of wall extensibility, can vary greatly among cells, from the highly directional (anisotropic) diffuse growth of elongating cells of stems and roots to the complex 2D growth patterns of jigsaw-puzzle-shaped epidermal cells of Arabidopsis (*Arabidopsis thaliana*) leaves, to localized growth gradients on the flanks of tip-growing root hairs and pollen tubes. Do the molecular mechanisms of surface expansion in these cases differ? What structural features make a wall extensible? Are there different molecular mechanisms of irreversible wall enlargement? How does the deposition of new wall material relate to the process of wall enlargement? How do the mechanical properties of the wall relate to its structure and its ability to extend irreversibly? We do not have consensus on the answers to most of these questions, but central to answering them is a deep understanding of cell wall structure.

## Update on cell wall components

What are the mechanical roles of the major cell wall components? What structural features make a wall extensible? To oversimplify a bit, the dominant structural polymers of the growing cell wall include cellulose, hemicelluloses, and pectins. These polysaccharides possess markedly different biochemical structures, conformations, and physical properties. Growing cell walls also contain small amounts of glycoproteins in various forms with diverse proposed functions (Marzol et al., 2018), but they are not considered here as their role in wall mechanics is uncertain. They are often called structural proteins because they lack enzymatic activity, but this does not mean they have a load-bearing role in the wall, an idea that has sometimes been proposed on little empirical evidence. An analogy is that of the walls in a house. Some are load bearing. These hold up the roof and bear the stresses arising from the weight of the roof. Others are not load bearing, but serve other functions. Likewise not all structural polymers in the cell wall bear its mechanical stresses.

I first present an update of the three dominant wall polysaccharides (cellulose, xyloglucan, and pectin) followed by concepts of how they may interact to make a strong yet extensible cell wall. Perspectives on these topics have changed considerably in the past decade.

### Cellulose

Chemically described as a linear chain of β1,4-linked D-glucose units, this polysaccharide sounds simple, yet its hierarchical organization, physical properties, and binding interactions lead to complex emergent properties and consequently many fundamental aspects of cellulose–matrix interactions in plant cell walls remain uncertain today. CMFs possess self-organizing behavior and structural subtleties with far-reaching consequences for cell wall assembly, mechanics, and growth. One would hardly guess this is so from the way cellulose is often represented in cartoons of the primary cell wall and consequently the nuances of cellulose’s properties are often overlooked.

**Figure 1:**
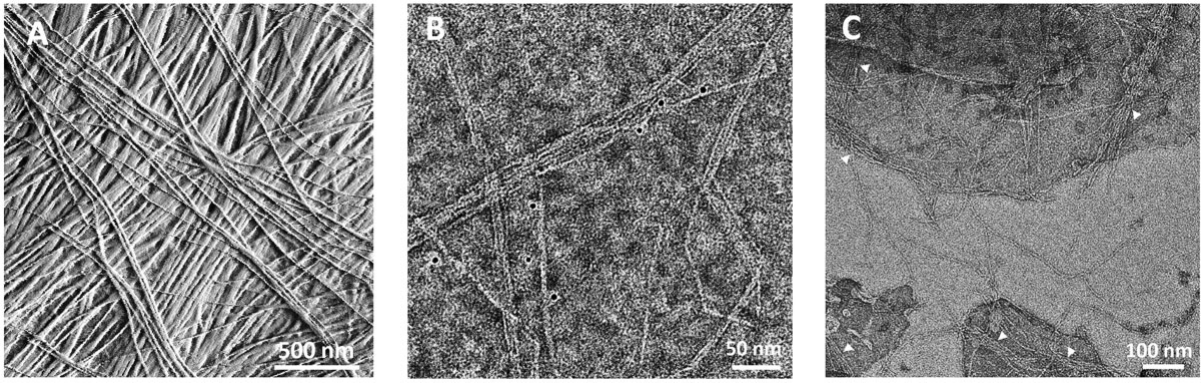
Images of bundling and aggregation by CMFs. A, AFM image of the surface of onion epidermal wall, showing extensive bundling and cross-ply construction (adapted from Zhang et al., 2014). B, Negative stained TEM of microfibrillated CMFs prepared from maize (*Zea mays*) primary cell wall by chemical and enzymatic treatments and mechanical shear and decorated with 5-nm nanogold particles conjugated to a cellulose-binding protein. Note extensive CMF bundling. The image is provided courtesy of Mark Frank and Liza Wilson. C, CMFs synthesized in vitro*.* Note they spontaneously form bundles and networked structures (indicated by arrow heads). The image is from Cho et al. (2017), used with permission.

Cellulose is synthesized in diverse fibrillar forms across the wide range of cellulose-synthesizing organisms, including bacteria, algae, tunicates, and plants (Tsekos, 1999; Wohlert et al., 2021). In land plants CMFs are synthesized by large cellulose synthase complexes (CSCs) embedded in the plasma membrane (reviewed by Allen et al., 2021). CMFs in plants are long (rough estimates of 1–10 μm are common) and thin (∼3 nm) and comprised many parallel glucan chains tightly packed into crystalline order (Nishiyama, 2009). Extensive hydrogen bonds between the chains stabilize the structure, but a larger contribution may come from London dispersion forces between the parallel chains (Nishiyama, 2018; Wohlert et al., 2021). Thus the remarkable stability and physical properties of the CMF arise from noncovalent interactions between glucan chains. For similar reasons, CMFs have a strong intrinsic tendency to aggregate laterally. In primary cell walls, this tendency can manifest as extensive 2D networks of bundled microfibrils in lamellae with distinctive microfibril orientations (Figure 1), while in many secondary cell walls cellulose may form a tightly packed 3D network of microfibril aggregates (“macrofibrils”) (Boyd and Foster, 1975; Terashima et al., 2004; Donaldson, 2007; Lyczakowski et al., 2019). These differences in cellulose organization likely result from different patterns of CMF deposition at the cell surface and from the presence of structurally different matrix polysaccharides that interact with cellulose in distinctive ways, modulating its self-assembly behavior and contributing to different mechanical behaviors (Atalla et al., 1993; Duchesne et al., 2001; Hult et al., 2003; Cosgrove and Jarvis, 2012; Meents et al., 2018; Terrett et al., 2019; Crowe et al., 2021). Additional research is needed to bring these inferences about the genesis and consequences of different cellulose organizations into a detailed mechanistic framework.

The strong tendency of cellulose to self-aggregate is readily observed in microfibrillated cellulose preparations, prepared by chemical pulping, mechanical shear, or in-vitro synthesis (Figure 1). For practical purposes such aggregation interferes with downstream processing (Eyholzer et al., 2010; Holland et al., 2019; Perzon et al., 2020; Trache et al., 2020). An effective way to reduce aggregation is by chemical oxidation of cellulose, yielding a surface decorated with carboxyl groups (Isogai et al., 2011). The charge repulsion reduces cellulose bundling in vitro and can partially reduce microfibril aggregation in complex cell walls (Zhang et al., 2016). The same principles apply to cellulose nanocrystals produced from cellulose pulps by limited hydrolysis with sulfuric acid (Ramires and Dufresne, 2011), which modifies cellulose surfaces with sulfate esters. The sulfated nanocrystals readily disperse as colloidal particles in water, but hydrolysis of the sulfate esters eliminates surface charge repulsion, leading to nanocrystal aggregation (Habibi et al., 2010; Moreau et al., 2016).

In native cell walls, strong noncovalent bonding between cellulose surfaces likely facilitates the formation of microfibril bundles and 2D networks, enabling layer-by-layer construction of the cross-lamellate (“cross-ply”) structure evident in many cell walls (Figure 1). At the newly deposited surface of onion epidermal walls, individual microfibrils are seen to merge into and out of bundle junctions, with most of the microfibril length, on average, aggregated in bundles of two or more microfibrils (Zhang et al., 2016). Such microscopic evidence is supported by estimates of extensive CMF bundling based on chemical oxidation of water-accessible surfaces (Shiga et al., 2021). As elaborated below, bundling enables direct transmission of tensile forces between CMFs without intermediation by the matrix (Zhang et al., 2021c). This cellulose organization may be important for wall extensibility, yet it is at odds with most contemporary depictions of the primary wall as a scaffold of well-separated microfibrils linked via hemicellulose tethers (Albersheim et al., 2011).

In many cases microtubules guide the direction of CSC movement at the cell surface, thereby guiding the orientation of CMFs (Paredez et al., 2006; Khan and Persson, 2020; Duncombe et al., 2022). In addition, preexisting CMFs may guide CSC movement (Baskin, 2001; Lloyd, 2011). Recent microscopic evidence for the latter concept comes from observations that autonomous CSCs (free of microtubule guidance) abruptly changed their direction of motion along the cell surface when they encountered the trail of another CSC and subsequently tracked along it (Chan and Coen, 2020). The nascent CMF was hypothesized to bind to a preexisting CMF, physically drawing the CSC in the new direction. Such entrainment of autonomous CSCs was proposed to amplify the guidance provided by cortical microtubules, reinforcing a common CMF orientation as a new lamella is assembled at the cell surface. In addition to guidance from microtubules and previously deposited CMFs, other unidentified factors may provide CSC guidance as well (Himmelspach et al., 2003; Xin et al., 2020).

In the outer epidermal wall of onion scales, CMFs in the most recently deposited lamellae were oriented coherently in a direction that changed abruptly in large steps from the underlying lamella, often roughly ±45° to the long axis of the cell (Zhang et al., 2014, 2016). This cross-ply pattern, which differs from the so-called helicoidal pattern formed by small rotational steps between successive lamellae, is also evident in many earlier studies of epidermal cell walls (Crowell et al., 2011) as well as in the thick walls of celery (*Apium graveolens*) collenchyma (Chen et al., 2019), but its origin is not entirely clear (Lloyd, 2011). Changes in CMF orientation may be connected to the rotation of microtubule arrays (Chan et al., 2010). This process may account for cases of regular (helicoidal) rotation of CMFs (Chan, 2012), but whether large, discrete steps in CMF orientation depend on rotation of microtubule arrays is uncertain (Lloyd, 2011). The origin of cross-ply cellulose patterns needs further study.

The cellulose organization in the cell wall is thus considered to be the result of the initial deposition pattern, presumably guided by cortical microtubules and reinforced by CMF self-assembly. The history of wall expansion may also influence cellulose of older layers through passive reorientation in the direction of growth (Preston, 1988; Anderson et al., 2010), but this may not always occur (Marga et al., 2005; Chen et al., 2019). Additionally, cellulose patterning is influenced by some extracellular proteins such as members of the COBRA-LIKE family (Liu et al., 2013; Sorek et al., 2014; Ben-Tov et al., 2018) and chitinase-like (CTL) proteins (Hauser et al., 1995; Sanchez-Rodriguez et al., 2012; Wu et al., 2012). Genetic disruption of these proteins disturbs normal cellulose organization and growth, but their mechanism of action is unclear. Mutational effects may be indirect, for instance by modulating ethylene biosynthesis, potentially a stress response to dysfunctional cell walls (Gu et al., 2019).

Cellulose organization may also be influenced by matrix polysaccharides in various ways. For instance, hemicellulose may function as a weak monomolecular adhesive between two microfibril surfaces to promote bundling or it may sterically hinder direct contact between CMF surfaces (Park and Cosgrove, 2012b; Zhao et al., 2014; Dammak et al., 2015). Biological evidence supporting this latter concept comes from the observation that cellulose is better aligned in the outer epidermal wall of an Arabidopsis mutant lacking xyloglucan (Xiao et al., 2016). A remarkably similar enhancement of CMF alignment was also observed in an Arabidopsis mutant defective in CELLULOSE SYNTHASE INTERACTIVE1 (CSI1; Xin et al., 2020), a protein that links CSCs to microtubules (Li et al., 2012). Without CSI1-mediated microtubule guidance, CMFs may be freer to self-organize. Similarly, treatment with oryzalin, a microtubule depolymerizing drug, resulted in a more parallel CMF organization without the normal cross-ply structure (Xin et al., 2020). When allowed to interact for a sufficient period of time in vitro, nanofibrillated cellulose likewise spontaneously packs into nearly parallel layers (Zhao et al., 2018). These results suggest that CMFs inherently tend to self-organize into roughly parallel alignment, when not steered otherwise.

Moving to a finer scale, the organization of glucan chains within the microfibril is an important structural determinant of the mechanical characteristics of CMFs as well as the surface properties governing their interactions with other CMFs and with matrix polysaccharides. Such interactions may be central to the assembly of a coherent, strong, and extensible cell wall. Key structural features of the CMF include the number of glucan chains, the geometry of their packing and their degree of order. For many years a CMF cross-section was believed to contain 36 parallel glucan chains, but more recent results favor 18 chains in the so-called elementary microfibril synthesized by the CSC. Measurements of CMF diameter in primary cell walls by AFM were consistent with 18-chain models (Zhang et al., 2016; Song et al., 2020), although uncertainties in the size and shape as measured by AFM make this a rough approximation at best; other AFM-based studies reported larger (Ding et al., 2014) and smaller (Niimura et al., 2010) diameters. Methods based on X-ray analysis, neutron scattering and solid-state nuclear magnetic resonance (SS-NMR) of cell walls generally estimate diameters larger than the equivalent of 18 chains (Fernandes et al., 2011; Newman et al., 2013; Wang and Hong, 2016; Phyo et al., 2017a), but these ensemble measurements include aggregates of two or more CMFs that may bias estimates to larger values; tightly bound hemicelluloses may also lead to overestimates of CMF diameters (Jarvis, 2018; Terrett et al., 2019; Thomas et al., 2020). Nonetheless, 36-chain models of CMFs continue to be discussed in many current research publications (Ciesielski et al., 2019; Zhou et al., 2020), particularly when the cellulose is sourced from wood pulp. Perhaps, there are natural variations in the cellulose-making machinery in plant cells that we do not yet appreciate and that result in varied CMF structures in different plant cell walls.

**Figure 2:**
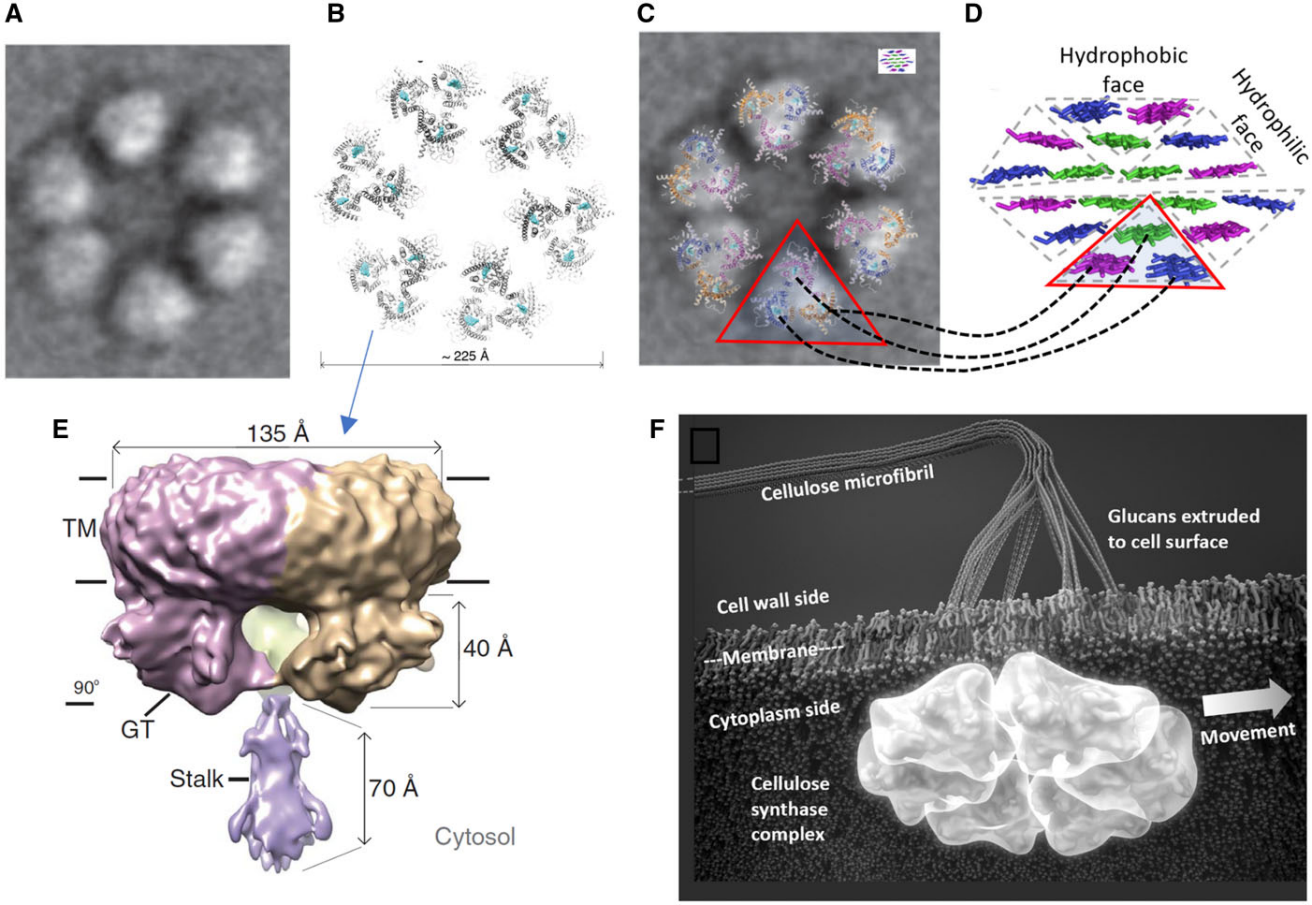
Cellulose is synthesized in land plants by a multimeric CSC. A, Hexameric organization of the CSC as seen by freeze-fracture electron microscopy. The image is ∼25 nm wide. B, Surface view of six trimer complexes of cellulose synthase transmembrane regions, arranged with hexagonal packing. C, Superposition of (A) and (B). Inset is a model of a CMF cross-section, drawn to scale with the CSC in the electron micrograph. The same CMF model is shown in (D) at larger scale; the red triangles in (C) and (D) are intended to illustrate the possible correspondence between the trimeric organization of the CSC and the organization of the CMF, with corresponding glucan chains indicated with dotted lines. E, Molecular model of a trimeric complex of cellulose synthase in side view. TM indicates the transmembrane region; GT indicates the glycosyltransferase region; the stalk structure from the three proteins likely extends into the cytoplasm to make contact with microtubules via linker proteins. F, Artistic rendition of a CSC embedded in a plasma membrane during the synthesis of a CMF. (A) From Nixon et al. (2016) used with permission; (B), (C), and (E) are from Purushotham et al. (2020), used with permission. (D) Adapted from Yang and Kubicki (2020) used with permission. (F) Adapted by Seong Kim from Vandavasi et al. (2016) used with permission.

The physical estimates of 18 chains in a CMF cross-section fall in line with implications from recent progress in the structure of plant cellulose synthase (CesA) and the CSC: (1) Images of six-lobed CSCs (“rosettes”) in native membranes, obtained by freeze-fracture transmission electron microscopy, were compared with computational models of CesA packed in different configurations (Nixon et al., 2016). The results supported a CSC with six hexagonally-packed subunits, each subunit containing three CesAs, making a total of 18 CesAs per CSC (Figure 2). Assuming each CesA made one glucan, the CSC product would contain 18 chains. (2) The cryo-electron microscopy (EM) structure of a homotrimer complex of poplar CesA8 provided atomic-level resolution of the enzyme structure and its packing in a structure that corresponds to one of the six subunits in a rosette CSC (Purushotham et al., 2020). A second homotrimer structure based on cotton (*Gossypium hirsutum*) CesA7 revealed an almost identical conformation (Zhang et al., 2021b).

The rosette model that emerges from these two studies predicts the positions of the 18 glucan chains as they emerge from the CSC, providing a starting point for physical modeling of how 18 chains may coalesce to form a crystalline CMF (Figure 2). We know little about this process. Based on modeling and EM images, Haigler et al. (2014) proposed that unstructured glucans may collect in a zone outside the CSC before being drawn into a crystallizing microfibril. This would provide a flexible hinge to allow the glucans to crystallize at right angles to the direction from which they emerge out of the CSC (Figure 2).

Eighteen chains can pack into a microfibril with various cross-sectional shapes. This shape in turn determines the extent of the microfibril surfaces lined with the nonpolar plane of the glucose ring or with polar hydroxyl groups extending from the sides of the glucose ring (Figure 2). These so-called hydrophobic and hydrophilic surfaces interact in distinctive ways with water (O'Neill et al., 2017; Trentin et al., 2021), xyloglucan (Zhao et al., 2014), other CMFs (Oehme et al., 2015; Sinko et al., 2015; Zhang et al., 2021a), and cellulose-binding proteins (Dagel et al., 2011; Liu et al., 2011) including expansins (Georgelis et al., 2012; Wang et al., 2013b). For these reasons, microfibril size and shape are important determinants of cell wall structure, assembly, the action of various proteins, and ultimately wall extensibility. Quantum modeling of various CMF shapes and comparisons with experimental ^13^C SS-NMR chemical shifts and X-ray diffractograms showed that two arrangements were nearly equivalent in their consistency between experimental and theoretical results, namely a 5-layer arrangement (34443) and a 6-layer arrangement (234432), where each integer designates the number of chains in a layer (Kubicki et al., 2018). Follow-up modeling gave a slight edge to the 6-layer (234432) arrangement and led to a proposal of how cellulose chains emerging from the CesA-trimers in the CSC may pack into a consolidated 18-chain microfibril as three-chain sectors (Yang and Kubicki, 2020; Figure 2). A detailed analysis of CMF shape based on AFM imaging likewise favored the 234432 model (Song et al., 2020).

In reality, these chain arrangements may vary because of crystallization defects, thermal noise, physical stresses, entrapment of matric polymers during microfibril formation, or lateral interactions with other wall components. Bending or stretching of the microfibril may induce chain rearrangements (Molnar et al., 2018; Ciesielski et al., 2019); so too may internal stresses within the microfibril, resulting from a slight twisting of the cellulose crystal (Zhao et al., 2013; Willhammar et al., 2021). Thus, the stacking arrangement of crystalline regions of the microfibril may be more varied than is suggested by these snapshots and could change further when two or more microfibrils come into contact, potentially melding crystalline regions (Newman et al., 2013). Such melded regions would hinder slippage between the melded CMFs, except at very high shear forces (Molnar et al., 2018).

Estimates of cellulose order within microfibrils indicate substantial chain disorder, varying with the specific method used to assess crystallinity (Park et al., 2010; Fernandes et al., 2011). The potential origins of cellulose disorder are various (Thomas et al., 2013). Surface chains interact with surface water and matrix polysaccharides and have fewer stabilizing interactions with internal chains. Additionally, there may be periodically disordered segments interspersed with highly crystalline regions. Spectroscopic and SS-NMR results were interpreted to mean that most of the disordered cellulose was on the surface of the microfibril, not in discrete segments along the microfibril (Fernandes et al., 2011; Wang et al., 2016; Wang and Hong, 2016). However, the data do not exclude the possibility of short segments of disordered cellulose along the microfibril. The production of cellulose nanocrystals of narrow size range by acid hydrolysis is considered to be evidence for periodic regions of disordered cellulose (Klemm et al., 2018; Trache et al., 2020). In primary cell walls, disorder regions may be sites of xyloglucan entrapment (Hayashi, 1989; Pauly et al., 1999; Park and Cosgrove, 2012b). Cellulose crystallinity in Arabidopsis hypocotyls was inversely associated with GR (Fujita et al., 2011); cellulose disorder likely influences binding of matrix polymers and frictional sliding of microfibrils during wall expansion, but clear evidence on these points seems to be lacking.

Another oft-discussed structural feature of the CMF is its tendency to twist, or not (Willhammar et al., 2021). This is potentially an important feature because it may influence lateral bonding of CMFs and formation of higher-order structures. Computer models predict CMF twisting, with various contributions ascribed to the chirality of cellulose or to its pattern of internal hydrogen bonding or even to influence of the force fields used in the modeling (Matthews et al., 2006, 2012; Hadden et al., 2013; Shklyaev et al., 2014; Bu et al., 2015; Kannam et al., 2017; Dumitrica, 2020). Twisting may generate internal stresses within the microfibril, building up along the length of the microfibril until relieved by disruption of crystalline order (Zhao et al., 2013). Recent electron diffraction studies of isolated nanocrystals of tunicate cellulose indicate twisting (Ogawa, 2019; Willhammar et al., 2021). However, tunicate cellulose is larger and more crystalline than cellulose from plant sources and the cellulose was highly processed and dried, potentially altering its structure.

Turning to plant celluloses, microscopic evidence of CMF twisting has been reported for woody cell walls after partial deconstruction with hot, dilute acid (Ciesielski et al., 2013). Ding et al. (2012) reported infrequent microfibril twisting in AFM images of dehydrated delignified secondary walls, but not in hydrated walls. They suggested that twisting was a drying artifact. AFM images of never-dried primary cell walls of onion did not show evidence of CMF twisting (Zhang et al., 2016). Recent additional evidence against CMF twisting in primary cell walls comes from use of a method called Grazing Incidence Wide Angle X-ray Scattering (Ye et al., 2020). The results indicated that cellulose crystallites have a preferred orientation relative to the plane of the cell wall. A preferred crystallite orientation would not be expected if CMFs twist along their axis. Such order might arise if CMFs formed 2D networks by lateral bonding of specific CMF surfaces. In Sugiyama et al. (1994), X-ray- and electron-diffraction analyses of the cross-lamellate walls of the alga *Valonia* indicated that these large CMFs do not twist. At this point, there seems little evidence of CMF twisting in native primary cell walls.

Finally, the CMF has very high flexural and tensile stiffness as a result of its chain packing density, order, and diameter. High flexural stiffness means the microfibril resists bending. In polymer physics, this property is measured as persistence length, which is the length over which chain direction randomizes during thermal motions. Judging from AFM images of surface-oxidized CMFs dried onto mica, persistence length was estimated to be 2.5 μm (Usov et al., 2015). This value may be an underestimate because of chemical damage to the cellulose during its isolation. Even so, this value is ∼200× greater than that of matrix polysaccharides. Cellulose is by far the least flexible component of the cell wall. The second characteristic of cellulose, its high tensile stiffness, is commonly estimated as 50–150 GPa (comparable to steel), meaning the microfibril strongly resists axial stretching. The CMF is often considered effectively inextensible within the context of normal cell wall stresses. Recent modeling indicated that CMFs in primary cell walls may stretch elastically up to ∼1% under the action of biologically relevant tensile forces, whereas the matrix polysaccharides are much more stretchy because of their ability to uncoil (Zhang et al., 2021c).

These physical properties, combined with strong lateral bonding between aligned CMFs, mean that cellulose potentially forms strong interconnected networks that determine the tensile mechanics of the primary cell wall. I will return to this point after considering xyloglucan and pectin.

### Xyloglucans

This group of structural polysaccharides constitutes the major hemicellulose in primary cell walls of most land plants (Scheller and Ulvskov, 2010; Park and Cosgrove, 2015), with the notable exception of grasses and related species where arabinoxylans predominate (Carpita, 1996; Pena et al., 2016). More rarely, xyloglucans also accumulate as extracellular storage polysaccharide in seeds of certain species such as tamarind (*Tamarindus indica*) and nasturtium (*Tropaeolum majus*; Kooiman, 1961; Buckeridge, 2010). Xyloglucan is tightly bound in the cell wall in most cases and requires strong alkali to solubilize it (Valent and Albersheim, 1974; Hayashi, 1989). This is taken as evidence that xyloglucan is hydrogen bonded to cellulose, although dispersion forces and hydrophobic effects potentially play a larger role in binding to cellulose in an aqueous environment (Lindman et al., 2021; Wohlert et al., 2021). Solubilized xyloglucan can re-bind to cellulose, but to a lesser extent than occurs in the native wall (Hayashi, 1989), perhaps because of physical entrapment by CMFs in native walls.

In its canonical form xyloglucan consists of a β-(1,4)-glucan backbone with a xylose attached to carbon-6 in 3/4 of the glucose units in a repeating backbone pattern (Schultink et al., 2014). O-acetylated galactose is frequently appended to the xylosyl residues and fucose may be further appended to some of the galactose residues, making branches of one, two, or three glycosyl units. Other sugars are included in some xyloglucans, varying by species and cell type (Muszynski et al., 2015). For instance, glucuronic acid is appended to some of the xylosyl residues in an unusual acidic xyloglucan found specifically in root hairs of Arabidopsis (Pena et al., 2012). A shorthand code to describe the different sidechains that decorate the glucan backbone is commonly used (Fry et al., 1993; Schultink et al., 2014).

Xyloglucans are synthesized by a series of glycosyl transferases in the Golgi (reviewed by Pauly and Keegstra, 2016) and are secreted via vesicles to the cell surface where they may bind to CMF surfaces and potentially affect local CMF organization. Little is known about this aspect of wall assembly or how secretion of cellulose and xyloglucan at the cell surface may be locally coordinated (Gu and Rasmussen, 2021). In vitro, adsorption of soluble xyloglucan onto cellulose nanocrystals was measured dynamically by quartz crystal microbalance (Villares et al., 2015), leading to a two-phase kinetic model of binding dynamics in which xyloglucan initially sticks to cellulose in a coiled configuration and subsequently rearranges itself into a flatter shape. In vivo, the space where xyloglucan and cellulose interact is much more crowded than in the dilute solution of in-vitro experiments; molecular crowding (Le Coeur et al., 2010) and steric conflicts in vivo may strongly affect the adsorption process, but this has not been studied to my knowledge.

In addition to physical adsorption, enzymatic ligation of xyloglucan chains by endotransglucosylases may contribute to xyloglucan integration into the cell wall and possibly to the production of xyloglucan hybrids with other wall polymers (Thompson et al., 1997; Rose et al., 2002; Hrmova et al., 2007; Simmons et al., 2015; Shinohara et al., 2017; Herburger et al., 2020; Stratilova et al., 2020a, 2020b). Long before the discovery of xyloglucan endotransglucosylase activity (Farkas et al., 1992; Fry et al., 1992; Nishitani and Tominaga, 1992), Albersheim (1975) speculated that enzymes with these activities might promote wall expansion; later researchers hypothesized both wall loosening and stiffening activities for these enzymes (Xu et al., 1995; Nishitani, 1998; Rose et al., 2002). However, in-vitro experimental tests of these ideas have detected little effect on wall mechanics (Saladie et al., 2006; Cosgrove, 2016) and knockout mutants display minor growth phenotype (Kaewthai et al., 2013), except under stress conditions (Ishida and Yokoyama, 2022). They are now commonly called “wall remodeling” enzymes, a description that avoids unconfirmed implications for wall mechanics and growth. Transglycosylases may account for xyloglucan–pectin hybrid molecules (Cornuault et al., 2014; Stratilova et al., 2020b) as well as other kinds of hybrids (Herburger et al., 2020), but their impact on wall mechanics is unclear.

Xyloglucan’s sidechains are important for its solubility in water (Sims et al., 1998) and its interactions with xyloglucan-modifying enzymes (Pena et al., 2004; Stratilova et al., 2020a). Sidechains modulate xyloglucan binding to cellulose in vitro (Lima et al., 2004; Chambat et al., 2005; Whitney et al., 2006; Stimpson et al., 2020), whereas in vivo no difference in tenacity of binding to cell walls was observed in the galactose-deficient xyloglucan of a *murus3* (mur3) mutant (Pena et al., 2004). This last study noted subtle hypocotyl phenotypes in the *mur3* mutant that were attributed to the requirement of the galactose residue for xyloglucan endotransglucosylase action to integrate xyloglucan into the cell wall, presumptively strengthening it. Sidechain structure may also influence the shape of xyloglucan, which takes on a swollen worm-like coiled conformation in dilute solution but aggregates at higher concentrations (Muller et al., 2011, 2013; Koziol et al., 2015). With a persistence length of ∼10 nm, xyloglucan is very flexible relative to cellulose, but somewhat stiff in comparison with simpler polymers.

**Figure 3:**
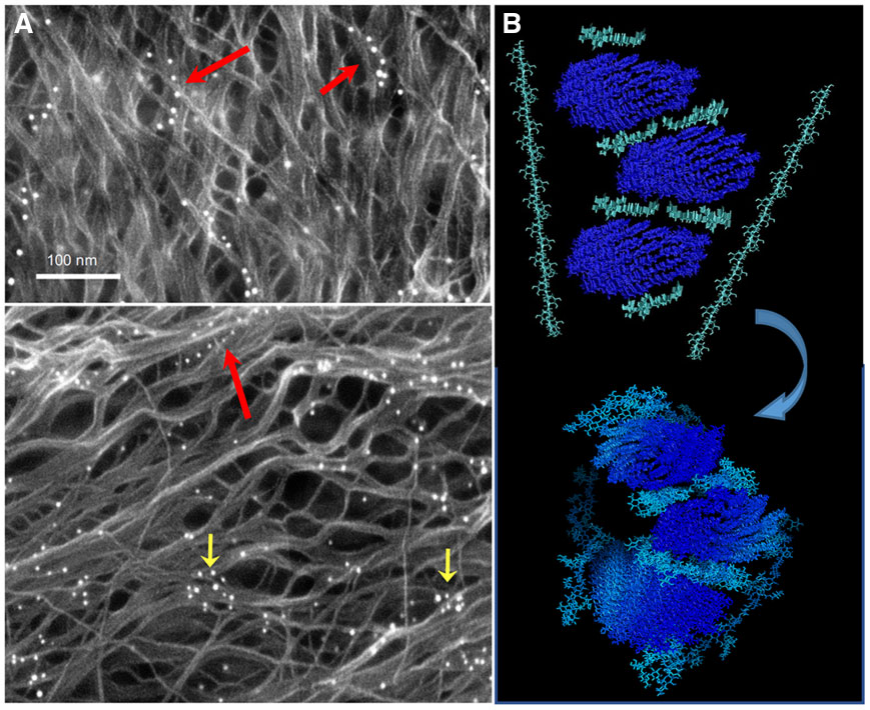
Xyloglucan location and conformation in relation to cellulose. A, Two scanning electron micrographs of the surface of onion epidermal cell walls labeled with nanogold particles conjugated to CBM76, a xyloglucan-binding protein. Nanogold appears bright white. The “string-of-pearls” (large red arrows) indicates xyloglucan in extended conformation; random clouds of nanogold indicate xyloglucan in swollen coiled conformation (small yellow arrows). HG was removed enzymatically before CBM76 labeling. Images from Zheng et al. (2018), used with permission. The 100-nm scale bar applies to both images. B, Molecular dynamics simulation of xyloglucan as a monomolecular adhesive between CMFs. (Top) End-on view of three CMFs (dark blue) initially positioned in parallel with six xyloglucan chains and with two xyloglucan chains positioned at right angles above and below the CMFs. (Bottom) After equilibration, xyloglucan chains bind to two cellulose surfaces, providing adhesive action. Image produced by Z. Zhao and D. Cosgrove, based on Zhao et al. (2014). For scale: the CMFs are ∼3-nm wide.

Since the 1970s cell wall cartoons have depicted xyloglucan as binding to CMF surfaces in an extended conformation, but with only circumstantial experimental support for this conformation (Vian et al., 1992; Pauly et al., 1999). This question was recently addressed by labeling of onion epidermal walls with Carbohydrate Binding Module 76 (CBM76) conjugated with nanogold (Zheng et al., 2018). CBM76 is a small xyloglucan-binding protein (Venditto et al., 2016). The wall surfaces were imaged by field-emission scanning electron microscopy (FESEM) with back-scattered electron detection, which allowed imaging of nanogold in the context of CMF organization (Figure 3). In comparison, conventional immunogold labeling of cell walls and imaging by transmission electron microscopy (TEM) distinguishes the nanogold very well but CMFs in wall sections are not well resolved and xyloglucan chain conformation is not evident (McCann et al., 1995; Freshour et al., 2003). In the FESEM study, nanogold patterns revealed xyloglucan in both extended and swollen random coil conformations (Zheng et al., 2018), indicating that some xyloglucan chains were well solvated (swollen coils) while other were bound in extended conformation (Figure 3). Xyloglucan may also be entrapped within CMFs or between bundled CMFs, but such xyloglucan regions would be inaccessible to nanogold labeling. SS-NMR studies detected few xyloglucan–cellulose interactions (Dick-Perez et al., 2011), indicating only small quantities of entrapped xyloglucan. Enzymatic digestion of pea (*Pisum sativum*) epicotyl cell walls indicated 14% of the xyloglucan (equal to 3% of the total wall) may be entrapped by cellulose (Pauly et al., 1999). Some of these may function as load-bearing junctions between CMFs, the “biomechanical hotspots” postulated by Park and Cosgrove (2012b) on the basis of the mechanical effects of endoglucanase digestions (see below).

How the natural variation in sidechains may influence the biological functions of xyloglucan is generally uncertain (Schultink et al., 2014; Park and Cosgrove, 2015). Mutation of XYLOGLUCAN-SPECIFIC GALACTURONOSYLTRANSFERASE1, which adds glucuronic acid to xyloglucan, resulted in shorter root hairs (Pena et al., 2012). These authors speculated that acidic xyloglucan may promote separation of CMFs in the growing root hair wall (the hemispherical tip), facilitating wall surface expansion during tip growth, but cellulose organization in the mutant root hair cell wall was not reported. TEM images of root hair cell walls suggest extensive CMF bundling (Houwink and Roelofsen, 1954). Schultink et al. (2013) found that the specific sugar attached to xylosyl residues may be changed genetically without loss of xyloglucan functionality, as judged by growth and cell wall creep of Arabidopsis stems. They concluded that the mere presence of a second sugar on the xylose residue may be more important than the substituent’s chemical identity (typically galactose in canonical xyloglucan). Kong et al. (2015) reported that Arabidopsis mutants deficient in xyloglucan galactosylation displayed a dwarf phenotype which could be restored by complete genetic removal of xyloglucan. Hence the dwarf phenotype may not arise from a deficiency in a structural role of xyloglucan, but from negative effects of a dysfunctional xyloglucan, perhaps disrupting the secretory system. This is an example where interpretation of a mutant phenotype in terms of cell wall function is not straightforward.

**Figure 4:**
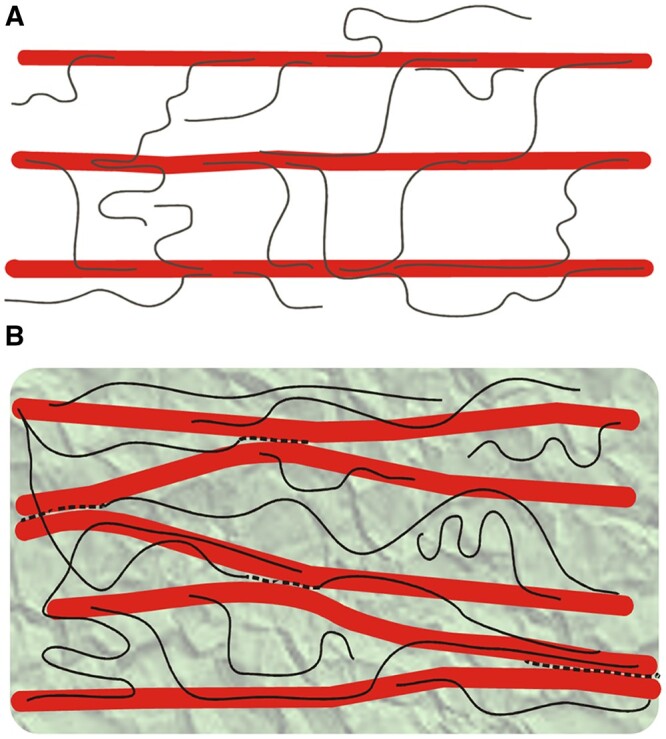
Cartoons of proposed arrangements of cellulose and xyloglucan. A, The tethered network concept shows CMFs (thick red rods) to be well spaced and mechanically linked by xyloglucans (thin black lines). B, The “biomechanical hotspot” concept proposes tight contacts between CMFs mediated in part by an amalgam of xyloglucan intermixed with disordered cellulose (black dotted lines). Pectins are shown as a gray-green background embedding the cellulose–xyloglucan network. Based on Park and Cosgrove (2012b).

What is the function of xyloglucan in the primary cell wall? For two decades the answer seemed clear: cell wall cartoons (“models”) depicted xyloglucan binding to cellulose surfaces in extended configuration and tethering microfibrils together (Figure 4; Albersheim et al., 2011). Mechanical tethering of CMFs by xyloglucan was widely accepted on scant empirical evidence. CMFs were depicted as well as separated from each other (no direct contacts, linked only by xyloglucan tethers), while pectins formed a hydrogel between the microfibrils. Such cartoons implicated xyloglucan in cell wall strength and perhaps for this reason the search for the mechanism of auxin-induced growth focused for many years on enzymatic modification of xyloglucans (Labavitch and Ray, 1974; Labavitch, 1981; Nishitani and Masuda, 1983; Fry, 1989; Hayashi, 1989; Talbott and Ray, 1992a). Given the widespread acceptance of this tethered network model, such a focus is logical, almost inevitable. Thus it came as a shock to the cell wall field when Cavalier et al. (2008) reported that genetic removal of xyloglucan in the Arabidopsis double mutant *xyloglucan xylosyltransferase 1* and *2* (*xxt1,xxt2*) resulted in only minor changes in aerial growth (*XXT* genes are required for xyloglucan synthesis). Root hairs in this and related xyloglucan-deficient mutants were short and malformed (Zabotina et al., 2012), but the general appearance of the rest of the plant was remarkably similar to wild-type. A similar conclusion emerged from a recent study of another set of xyloglucan mutants with defects in the five *Cellulose Synthase Like-C* genes responsible for the synthesis of xyloglucan’s backbone (Kim et al., 2020). In another study, xyloglucan deficiency did not visibly affect cell wall regeneration in Arabidopsis protoplasts (Kuki et al., 2020). These results undermine the concept that xyloglucan functions as a major load-bearing tether between CMFs.

For the *xxt1,xxt2* mutant, stretching assays showed that cell walls were more compliant (less stiff) in the mutant compared with wild-type walls (Cavalier et al., 2008; Park and Cosgrove, 2012a). At face value, this result suggests a wall stiffening role for xyloglucan, but this simple interpretation is confounded by subsequent observations that CMFs were better aligned with each other in the mutant and were oriented more transverse to the long axis of the cell (Xiao et al., 2016). Because transverse microfibrils offer little resistance to axial stretching (Zhang et al., 2021c), the change in cellulose orientation likely accounts for the increased compliance of the walls lacking xyloglucan. An increased brittleness (Sowinski et al., 2022) may also arise from altered cellulose organization. This is another example in which the facile interpretation of a wall phenotype may be misleading.

The greater CMF alignment in *xxt1,xxt2* walls suggests that xyloglucan interferes with cellulose bundling but subtle changes in mechanical signaling between the cell wall and the cytoskeleton may also be involved. Microscopy of living *xxt1,xxt2* hypocotyl cells revealed microtubules to be less stable than in wild-type (Xiao et al., 2016), perhaps contributing to altered hypocotyl curvatures in the mutant (Aryal et al., 2020; Jonsson et al., 2021; Velasquez et al., 2021). Subtle phenotypes also occurred in the shoot apical meristem, including changes in meristem shape, microtubule patterning, and exaggerated microtubule instabilities (Zhao et al., 2019). Sowinski et al. (2022) documented increases in pectin and glucomannan in *xxt1,xxt2* hypocotyls, but negligible changes in leaves. In evaluating xyloglucan mutants, it is difficult to disentangle direct mechanical effects of the xyloglucan deficiency from indirect or pleiotropic effects. This is likely to be true of other cell wall mutants. Complementary approaches are needed.

Taking a different tack, we tested the proposed tethering role of xyloglucan by digesting isolated cell walls with xyloglucanase and assessing effects on cell wall mechanics. Xyloglucan digestion had negligible biomechanical effects in walls from cucumber (*Cucumis sativus*) hypocotyls (Park and Cosgrove, 2012b), Arabidopsis hypocotyls (Park and Cosgrove, 2012a), and onion epidermis (Zhang et al., 2019), indicating xyloglucans do not function as mechanical tethers between CMFs. This direct approach is not confounded by potential compensatory alterations of wall structure, as may occur in genetic mutants.

On the other hand, large biomechanical effects were found when cell walls were digested with bifunctional endoglucanases that hydrolyze *both* xyloglucan and disordered cellulose, such as cellulase family-12A (Cel12A) from *Trichoderma reesei* (Park and Cosgrove, 2012b). These enzymes increased the axial compliance of hypocotyl walls and induced a time-dependent extension (creep) of walls held at constant force. Perplexingly, combining a xyloglucan-specific endoglucanase with a cellulose-specific endoglucanase—to mimic Cel12A’s bifunctional hydrolytic activity—failed to produce these biomechanical actions. Also, Cel12A-induced cell wall creep only after a long lag, varying from 40 min at low concentration to 6 min at saturating concentrations of Cel12A (Yuan et al., 2001). Six minutes is a long time in terms of enzymatic action. In comparison, exogenous addition of α-expansin stimulated cell wall creep in similar experiments within seconds (McQueen-Mason et al., 1992). The results of these enzyme digestions run contrary to the hypothesis that xyloglucan acts as a mechanical tether between CMFs, but they do implicate a mechanical role for a minor xyloglucan component in regions of limited enzyme accessibility. These regions, dubbed “biomechanical hotspots,” were hypothesized to be tight xyloglucan-mediated junctions between CMFs (Park and Cosgrove, 2012b; Figure 4). They may be similar to cellulose-entrapped xyloglucans detected in pea epicotyl walls by Pauly et al. (1999).

Related points: As noted above, cell walls of the *xxt1,xxt2* line were more compliant in stretching assays compared with wild-type, yet shoot growth was slightly reduced (Park and Cosgrove, 2012a; Xiao et al., 2016; Sowinski et al., 2022). Epidermal walls of the *xxt1,xxt2* line lost their normal cross-ply structure (Xiao et al., 2016) and exhibited a diminished “acid growth” response (Xin et al., 2020), which is mediated by endogenous α-expansins (Cosgrove, 2000). They were also less responsive to exogenous α-expansin (Park and Cosgrove, 2012a). Connecting and extending these observations, Xin et al. (2020) discovered that diminished acid growth is common to other instances lacking the typical cross-ply organization in epidermal walls. This was the case in hypocotyls treated with oryzalin (a microtubule depolymerizing drag) and in the *csi1* mutant, as well as in the *xxt1,xxt2* mutant. Why walls lacking a cross-ply construction have reduced acid growth is uncertain but the results point out another example of the disparity between mechanical compliance and growth extensibility. A related phenomenon is seen in the *mur3* xyloglucan mutant (above) where the mechanical strength of the hypocotyl was substantially reduced, yet its elongation was not affected (Pena et al., 2004). These results illustrate the point that mechanical compliance and growth extensibility are not necessarily linked.

To summarize, xyloglucan binds to cellulose and may influence CMF self-assembly in multiple ways, but the long-held hypothesis that xyloglucans mechanically tether CMFs seems dubious to me. It is time to reconsider xyloglucan’s functions with a fresh eye. Aberrant root hair growth and subtle developmental effects in xyloglucan-deficient mutants may originate partly from effects on cytoskeletal dynamics. The mechanical action of xyloglucan-cutting enzymes on cell walls in vitro suggests that an amalgam of disordered cellulose and xyloglucan may bind CMFs together in limited junctions (“biomechanical hotspots”), proposed as potential sites of wall loosening by α-expansin (Park and Cosgrove, 2012b), but the structure, origin, and distribution of these hypothetical junctions need more detailed characterization.

### Pectins

Chemically, the most complex of the major cell wall polysaccharides, pectins comprise ∼30% to >50% of the polysaccharide in many primary cell walls (McNeil et al., 1984; Caffall and Mohnen, 2009; Ropartz and Ralet, 2020). Grass cell walls present an exception to this statement, as they generally contain more arabinoxylan and cellulose than pectin (Burke et al., 1974; Carpita, 1996; Carpita et al., 2001). The dazzling diversity of complex chemical structures and network-forming mechanisms of pectic polysaccharides, combined with their high water-holding capacity, enables many possible hydrogel structures, a gold mine of soft materials with tunable physical properties for use in food processing and other applications (Williams, 2020; Zdunek et al., 2021). It is widely believed that the water-holding capacity of pectic polysaccharides influences the physical properties and extensibility of primary cell walls (Jarvis, 2002). However, the physical state of pectin in native walls is not well defined because tools for characterizing higher-order pectic structures within complex walls are limited; the major tools include ^13^C SS-NMR (Foster et al., 1996; Ha et al., 1996; Wang et al., 2012; Temple et al., 2021), optical microscopy using fluorescent probes for pectins (Rydahl et al., 2018; Voiniciuc et al., 2018b) and scanning electron microscopy (Domozych et al., 2014; Zhang et al., 2016). Current views on the potential roles of pectins in plant morphogenesis and growth are extraordinarily diverse and have been the subject of numerous reviews (Palin and Geitmann, 2012; Saffer, 2018; Anderson and Kieber, 2020; Yang and Anderson, 2020; Haas et al., 2021; Shin et al., 2021). In this section, I summarize this complex and contradictory subject with regard to mechanisms of wall enlargement.

Pectins are galacturonic acid-rich polysaccharides comprising two major domains: homogalacturonan (HG) and rhamnogalacturonan-I (RG-I), plus a small but complex domain (RG-II) that is often linked to HG (Caffall and Mohnen, 2009; Ropartz and Ralet, 2020). Arabinans and galactans (both neutral polysaccharides) are also considered pectic polysaccharides because they are often (though not always) covalently linked to the RG-I backbone. Pectic domains are believed to be covalently linked together to some extent, varying with wall sample, and there is evidence of pectin/hemicellulose hybrids in some cells (Popper and Fry, 2008; Tan et al., 2013; Cornuault et al., 2014), but not all (Talbott and Ray, 1992b). Unlike xyloglucan or cellulose, a substantial proportion of pectic polysaccharide may be solubilized from cell walls by hot water or weak phosphate buffer, for example, Zablackis et al. (1995), indicating weak binding to wall components.

HG is a linear polymer of α-(1-4)-linked galacturonic acid constituting ∼65% of the pectic polysaccharide in many primary walls. When HG is secreted to the growing cell wall, most of its carboxyl groups are methyl esterified. The methyl groups may subsequently be hydrolyzed by pectin methylesterase (PME), which is most active at pH 7–8. The resulting charge repulsion between –COO^−^ groups can swell the cell wall, increasing its hydration (Wang et al., 2020). This is in keeping with the view that pectins are the major determinants of primary cell wall thickness (Jarvis, 1992). The negative charges may also induce spatial redistribution of charged proteins and ions to different microdomains within the wall (Moustacas et al., 1991; Pilling et al., 2004; Hocq et al., 2017) and potentially modify the activity of transporters, receptor kinases, and other proteins embedded in the plasma membrane.

In vitro, pectins form physical networks and hydrogels by a variety of physico-chemical mechanisms (Jarvis, 2002; Zsivanovits et al., 2004; Williams, 2020; Pieczywek et al., 2021; Zdunek et al., 2021), a property that has drawn considerable attention by food chemists (Gawkowska et al., 2018). Within native cell walls, the physical state of pectins—intermixed as they are with cellulose, xyloglucan, glycoproteins and other polymers—is not so clear, and it is uncertain whether they undergo gel–sol transitions characterized in pure HG isolates (Ngouemazong et al., 2012). Such transitions can be measured as endothermic events by differential scanning calorimetry (Iijima et al., 2021), but there seems to be only a single report of such a potential transition in growing cell walls (Lin et al., 1991). In pectin-rich primary cell walls from apple (*Malus pumila*) fruit, no evidence of gel–sol transition was detected (Aguilera et al., 1998). In pectin-rich regions nearly devoid of cellulose, such as the middle lamella, especially at tricellular junctions (Willats et al., 2001), pectins may assume different conformations and states than in regions rich in cellulose and hemicellulose. Moore and Staehelin (1988) reported that most of the immunogold labeling of RG-I was localized to the middle lamella, while xyloglucan was detected in the cellulose region. Dynamic changes in the physical and chemical state of pectin is a subject of numerous hypotheses and speculation about microdomains with distinctive properties (Hocq et al., 2017; Haas et al., 2020, 2021).

Within the cell wall, at least three mechanisms are proposed to contribute to the formation of HG networks and to stabilize pectins: (1) covalent crosslinking of RG-II by borate (O'Neill et al., 2004); (2) lateral aggregation of highly methylesterified HG chains at low pH (Hernandez-Cerdan et al., 2018; Pieczywek et al., 2021); and (3) calcium (Ca^2+^)-mediated junctions of de-esterified HG (Cao et al., 2020). These so-called “egg-box” junctions require a continuous block of ∼10 de-esterified residues (estimates vary) as well as available Ca^2+^ (Powell et al., 1982; Zdunek et al., 2021).

The relative importance of these three networking mechanisms for the mechanical extensibility of growing cell walls is unclear. Arabidopsis mutants defective in RG-II crosslinking produced smaller rosettes (O'Neill et al., 2001), a phenotype suggestive of reduced wall extensibility despite reduced pectic crosslinking. As a confounding effect, wall integrity defects in these mutants may initiate secondary responses that inhibit cell growth (Sechet et al., 2018). Removal of wall Ca^2+^ by chelators or low pH led to HG solubilization (Kobayashi et al., 1999), cell wall swelling (MacDougall et al., 2001), and cell wall softening as measured by tensile testing (Virk and Cleland, 1990) and by surface indentation (Wang et al., 2020). Such results point to substantial Ca^2+^-mediated condensation of HG in primary cell walls, potentially influencing wall mechanics. Because HG is secreted by growing cells in a highly methylesterified form incapable of forming egg-box junctions, formation of egg-box type junctions requires processive PME action and a Ca^2+^ supply. Egg-box structures have a distinctive SS-NMR signature (Jarvis and Apperley, 1995). This signature was detected as a minor signal in wild-type Arabidopsis cell walls while cell walls of a mutant defective in HG methyl esterification displayed increased signal (Temple et al., 2021). The role of egg-box junctions in normal cell walls remains an open question (Hocq et al., 2017).

Enzymatic de-esterification of HG has drawn renewed interest in recent years on several fronts, from plant development to defense. Plant PME activity depends on pH, with highest activity at pH 7–8, falling off at acidic pH (Jolie et al., 2010; Senechal et al., 2015). The *PME* gene family in Arabidopsis encodes 66 predicted proteins whose enzymatic activities are largely uncharacterized (Pelloux et al., 2007), with the notable exception of AtPME3 (Senechal et al., 2015). At pH 7.5 recombinant AtPME3 acted processively on methylesterified HG in vitro to produce blocks of de-esterified residues whereas at pH 4 its activity and processivity were strongly reduced. PME activity is also reduced by PME inhibitor (PMEI) proteins, encoded by a gene family of 71 members in Arabidopsis (Wang et al., 2013a). PMEIs can bind PMEs, potentially with pair-wise protein specificity (Wormit and Usadel, 2018). AtPMEI7 bound AtPME3 in vitro in a pH-dependent manner, forming an inactive complex at pH 5, while binding and inhibition were reduced at pH 7.5 (Senechal et al., 2015). PMEIs are proposed to modulate PME activities in a wide variety of biological contexts with proposed roles in cell adhesion, mechanics, development, wall stress signaling, and defense against pathogens (Lionetti et al., 2017; Liu et al., 2018; Wormit and Usadel, 2018).

Our understanding of the potential wall-stiffening effects of pectin de-esterification has become muddled in recent years. The conventional view, based on extensive in-vitro results with HG gels, is that PME action leads to pectic gel stiffening by formation of Ca^2+^-mediated junctions (Williams, 2020). This concept arose largely from experiments with isolated pectins and has been extrapolated to the native cell wall, but evidence that such junctions actually exist and reduce the mechanical extensibility of native cell walls is limited, contradictory, or based on associations [reviewed by Wang et al., 2020). In mung bean (*Vigna radiata*) hypocotyls, poplar (*Populus sp*.) cambium and suspension-cultured cells of flax (*Linum usitatissimum*), pectins were more highly methyl-esterified in growing cells than in inactive cells (Goldberg et al., 1996). In pollen tube walls, local cell stiffness as measured by surface indentation was greatest in regions rich in de-esterified HG as gaged by antibodies (Zerzour et al., 2009; Chebli et al., 2012). These results are consistent with the concept that PME action leads to a stiffer cell wall. In contrast, surface indentation studies of the shoot apical meristem of Arabidopsis found the opposite association, with reduced stiffness in regions rich in de-esterified HG (Peaucelle et al., 2011). Likewise, in Arabidopsis hypocotyls, Peaucelle et al. (2015) reported that HG de-esterification corresponded to softer surfaces. However, the opposite trend was reported by Bou Daher et al. (2018) who found that induction of AtPME5 in hypocotyls increased indentation stiffness while induction of the inhibitory PMEI13 decreased stiffness. Thus, the mechanical effects of HG de-esterification in vivo, as measured by tissue indentation, appear to be variable. In a review of this topic, Bidhendi and Geitmann (2016) concluded that assessment of the degree of pectin methylesterification is not a reliable proxy for mechanical behavior.

Interpretations of these results for conclusions about wall extensibility are complicated by two issues. First, changes in pectin esterification in living cells may activate wall integrity responses, as described below, which could alter wall mechanics by a variety of secondary responses and thus confound the direct mechanical effects of de-esterification. Second, it is often assumed that indentation stiffness corresponds to tensile stiffness of the wall. This may not be a safe assumption (Box 1). With these two issues in mind, we compared the mechanical effects of PME treatment of isolated epidermal walls as measured by indentation and tensile stretching (Wang et al., 2020). Use of native but nonliving cell walls avoided complications from biological responses to PME action. Without added Ca^2+^, PME treatment softened the cell wall, as judged by indentation (modulus reduced by ∼70%). Parallel tensile measurements showed that PME treatment did not alter elastic stiffness, but did increase plasticity slightly, by ∼20%. Thus, PME-induced changes in indentation and tensile mechanics were quite different. Wall thickness also increased after PME treatment, a result of increases in wall electrostatic potential, charge repulsion, and hydration. The increased hydration likely reduced the wall’s resistance to indentation. However, despite this softening action, PME treatment did not induce cell wall creep (time-dependent extension of the wall when held at constant force). Thus, while PME action in the absence of exogenous Ca^2+^ softened the cell wall by some measures, it did not loosen the wall as judged by creep experiments. Moreover, acid-induced wall extension by endogenous α-expansins decreased by ∼50% following PME treatment, despite PME’s wall-softening effect. This study illustrates the fact that different metrics of wall mechanics do not change coordinately. A similar conclusion was reached by Zhang et al. (2019) using other enzyme treatments (Box 1). A likely explanation is that wall polymer motions during indentation differ from those during tensile stretching, as a consequence of the wall’s anisotropic structure, but the time and length scales of the assays may also factor into the different mechanical responses.

Our understanding of pectin conformation and interactions within the wall is still in its infancy. AFM and TEM images of isolated pectic fragments dried onto surfaces displayed a variety of shapes, including rods, branched chains, and helical structures (Round et al., 2010; Domozych et al., 2014; Williams et al., 2020; Pieczywek et al., 2020a). In contrast, pectins on the surface of native onion walls formed a porous polygonal network as visualized by AFM and FESEM (Zhang et al., 2016). When hydrated, these surface chains were so flexible they were not detected in AFM scans which imaged only the microfibrils below the surface pectin layer. The AFM tip passed right through the soft pectins. When the pectins were stiffened by Ca^2+^ addition or by dehydration, they became visible by AFM as coiled strands. In a different study that combined immunolabeling with super-resolution fluorescence microscopy, pectins were detected as diffuse bands on the surface of anticlinal walls of Arabidopsis leaf epidermis (Haas et al., 2020). The fluorescence pattern was interpreted as arrays of long, 40-nm wide HG filaments. On the cell surface of the alga *Penium margaritaceum*, tightly packed strands of HG and RG-I form an intricate lattice that becomes a punctate pattern after PME treatment (Domozych et al., 2014). The different shapes reported in these studies highlight the need for further analysis of HG conformation in native cell walls.

Turning to RG-I, this complex polysaccharide consists of a backbone of alternating rhamnose and galacturonic acid residues variably decorated with complex side chains composed predominantly of galactose and arabinose residues, mostly attached to the backbone rhamnose units (McNeil et al., 1984). The sidechain structures are diverse and vary by tissue and species (Oechslin et al., 2003; Mikshina et al., 2015). Structurally diverse forms of RG-I may be present in a single cell type (Lee et al., 2013). Particularly, striking images of “bottle brush” structures were observed in seed mucilage extracted from the Arabidopsis *bifunctional b-D-xylosidase/a-L-arabinofuranosidase1* (*bxl1-3*) mutant (Williams et al., 2020). This mutant is defective in an enzyme that trims arabinan chains (Arsovski et al., 2009), resulting in RG-I with exceptionally long arabinan sidechains. The bottle brush structures were interpreted as helically wound chains of RG-I with regularly spaced arabinan side chains that were much longer in the *bxl1-3* mutant compared with wild-type. In potato (*Solanum tuberosum*) tuber, immunogold localization by TEM indicates that the galactan and arabinan sidechains of RG-I are dispersed within the primary cell wall and at cell edges (Oomen et al., 2002), but additional information on RG-I structure is not evident in the micrographs. The arabinan and galactan epitopes largely disappeared when rhamnogalacturonase was ectopically expressed in the Golgi, suggesting that RG-I anchors these neutral chains in the cell wall. In transgenic potato tuber with various RG-I modifications, growth was not visibly different from wild-type tubers but compression analysis of tuber slices revealed increased brittleness (Ulvskov et al., 2005). Likewise, enzymatic truncation of galactans in Arabidopsis resulted in only minor changes in growth (Obro et al., 2009). Transgenic poplar with ectopic expression of RG-lyase showed increased cell separation during wood processing, supporting a role for RG-I in cell adhesion (Yang et al., 2020). Such a role might account for the brittleness results of Ulvskov et al. (2005) in potato tuber and is consistent with RG-I localization to the middle lamella (Moore and Staehelin, 1988). Thus, RG-I appears to function in cell wall adhesion but there is little evidence for a major direct role in wall extensibility or cellulose organization.

Assessments of pectin–cellulose interactions have been informed by several experimental approaches (Gawkowska et al., 2018). HG in solution displayed little capacity to bind cellulose, while galactans and arabinans showed moderate binding, although appreciably less than xyloglucan (Zykwinska et al., 2005, 2008a, 2008b). Negligible direct binding of HG to cellulose is consistent with results of Agoda-Tandjawa et al. (2012) who found that addition of low-methoxy pectin did not change the rheology of microfibrillated cellulose suspensions. Consistent with this report, small-deformation rheology of ground cell walls was largely attributed to cellulose–cellulose interactions with only a secondary contribution from pectin and other matrix polysaccharides (Whitney et al., 1999).

Similar inferences about weak pectin–cellulose interactions were reached in studies in which pectin was added to *Gluconacetobacter xylinus* cultures producing cellulose pellicles (Chanliaud and Gidley, 1999; Park et al., 2014; Lin et al., 2016; Lopez-Sanchez et al., 2016, 2017), although when pectins were gelled with added Ca^2+^ there were more complicated behaviors. HG binding to cellulose in vitro was detected by quartz crystal microbalance, a very sensitive method; binding quickly reversed with water washes, indicating weak, reversible binding. Xyloglucan, in contrast, bound tightly and irreversibly to cellulose in similar studies (Benselfelt et al., 2016; Villares et al., 2017). In ^13^C SS-NMR studies of Arabidopsis cell walls, abundant cellulose-HG cross-peaks were detected (Wang et al., 2012, 2015). To be clear, the cross-peaks measure proximity, not binding strength. Pectins in the wall were more dynamic than cellulose (Phyo et al., 2017a, 2017b, 2018), which is consistent with weak binding between these two polymers. These studies indicate extensive, but weak, noncovalent binding interactions between cellulose and HG.

Other studies based on extractability of pectins from cell walls suggest strong association or entrapment of RG-I with cellulose in some cases. After sequential extraction of primary cell walls from apple fruit, RG-I with diverse sidechains remained in the cellulose residue (Oechslin et al., 2003). A specific interaction with cellulose was not established, but noncovalent entrapment and covalent linkages were considered possibilities. In a similar approach with carrot (*Daucus carota*) cell walls, Broxterman and Schols (2018) found that RG-I was resistant to 6-M alkali extraction (rather harsh conditions that swell cellulose), yet was released by treatment with a mixture of endo- and exo-glucanases. The authors proposed a covalent, but undefined, linkage between cellulose and RG-I sidechains, but physical entrapment also seems possible. Among the three different cell walls tested in this study, the stable complex was unique to carrot. In an analysis of cell wall polysaccharides of Arabidopsis leaves, Zablackis et al. (1995) likewise found that RG-I was released by cellulase treatment, although they did not comment on it. In the seed coat of Arabidopsis, RG-I contains a xylan chain that is proposed to anchor the pectic mucilage to the surface of cellulose (Hu et al., 2016; Ralet et al., 2016). Does a similar mechanism function in growing cell walls to anchor RG-I to cellulose?

While xyloglucans can be deleted genetically with only minor phenotypic effects on Arabidopsis growth, comparable mutants in the pectin realm have not yet been identified (Atmodjo et al., 2013; Biswal et al., 2018a; Voiniciuc et al., 2018a), perhaps because such mutations would be lethal. As described above, reduction of RG-I sidechains by ectopic expression of hydrolytic enzymes yielded plants with only minor growth phenotypes. Ectopic expression of fungal polygalacturonase in tobacco (*Nicotiana tabacum*) and Arabidopsis led to a slightly reduced HG content and stunted growth (Capodicasa et al., 2004). The reduced growth does not support the idea that polygalacturonase has wall loosening activity, but the opposite inference was obtained for a polygalacturonase-deficient mutant of Arabidopsis, which displayed reduced leaf growth (Rui et al., 2017). Overexpression of a polygalacturonase gene in Arabidopsis by activation tagging resulted in larger rosette leaves, early stem lignification and stem stiffening (Xiao et al., 2017). In other studies, changes in pectin biosynthetic enzymes led to unexplained changes in various wall components, stunted growth, and other perplexing responses: Du et al. (2020) found aberrant cellulose organization in an Arabidopsis mutant (*quasimodo2*) defective in a pectin methyl transferase gene; overexpression of GALACTURONOSYLTRANSFERASE12 (GAUT12), a putative pectin biosynthesis gene, decreased poplar growth by half (Biswal et al., 2018b); reduced expression of the pectin biosynthesis gene GAUT4 led to increased growth in switchgrass (*Panicum virgatum*) and decreased recalcitrance for biomass processing (Biswal et al., 2018a).

The basis for these varied and discordant phenotypes in pectin-related mutants is unclear. One possibility is that pectins are involved in normal cellulose assembly, perhaps by modulating CMF bundling. This is a case where in-vitro experiments of self-assembly of nanofibrillated cellulose might give insights (Zhao et al., 2018). Another possibility is that pectin defects activate wall integrity signaling pathways, eliciting cellular responses that modify growth and wall structure (Franck et al., 2018; Vaahtera et al., 2019). Pectins are strongly bound to WALL-ASSOCIATED KINASEs (Kohorn et al., 2014) and to other surface receptor kinases such as FERONIA (Doblas et al., 2018; Feng et al., 2018; Franck et al., 2018; Lin et al., 2022) which initiate cellular changes in pH, Ca^2+^, signaling by reactive oxygen species, RALF peptides and nitric oxide (Shih et al., 2014; Duan et al., 2020; Zhang et al., 2020). These responses are proposed to be part of an autocrine feedback system to limit growth-related cell wall damage associated with pectin modifications (Wolf et al., 2012, 2014; Hofte, 2015; Doblas et al., 2018). Whether substantial cell wall damage is inherent to normal cell growth has not been established, to my knowledge, although reactive oxygen species may chemically degrade wall polysaccharides (Fry et al., 2001; Tenhaken, 2015). Genetic or pharmacological alteration of pectin methyl-esterification can also activate brassinosteroid signaling, thereby altering expression of many genes directly or through cross-talk with other hormone pathways, potentially modifying the wall with far-reaching consequences (Wolf et al., 2012, 2014; Hofte, 2015). Whatever the mechanism, downstream changes in cellulose organization may have large mechanical consequences, confounding assessments of the direct mechanical contribution of pectin.

Considering the foregoing results, pectin–cellulose interactions in cell walls may be summarized as follows: HG binds very weakly to cellulose in vitro, yet makes extensive contacts with cellulose in the native cell wall; arabinan and galactan side chains of RG-I may bind to cellulose with moderate strength in vitro, but further work is needed to assess their importance for wall structure or mechanical properties in growing walls. In the unusual mucilage-rich secondary cell walls of the Arabidopsis seed coat, a xylan may link RG-I to cellulose.

Following these summaries of the physical properties of the major classes of wall polysaccharides, we turn now to the issue of how their arrangement makes for an extensible cell wall.

## Cell wall models: relating structure to mechanics and polymer motions

When plant biologists think of cell wall models, they generally think of 2D or 3D sketches of the placement, conformation, and connections of the various components of a cell wall. Sometimes these sketches are little more than a graphic parts list, but the most useful depictions include implicit predictions about the molecular basis of wall structure, mechanics, and potential mechanisms for modulating cell wall growth. Thus the tethered network model, a textbook staple for decades (Albersheim et al., 2011), depicts well-separated CMFs connected by xyloglucan tethers (Figure 4), implying that the xyloglucan backbone transmits tensile forces between microfibrils and that wall expansive growth requires cutting or shifting of xyloglucan tethers (Passioura and Fry, 1992). To test this concept, we digested primary cell walls with xyloglucanase and looked for evidence of mechanical weakening (Park and Cosgrove, 2012b). The model flunked this test, sending us back to the drawing board to account for these and other results at odds with what was then the conventional picture of the growing cell wall. The “biomechanical hotspot” concept emerged, positing a key structural role for cellulose–cellulose junctions, in some cases bonded by an amalgam of xyloglucan and disordered cellulose chains (Figure 4).

Diagrams such as these can convey concepts at a glance but they lack the explicit physical framework necessary to generate quantitative predictions for comparison with experimental results. Many such graphical “models” have been published, beginning perhaps with the sketch of parallel, well-spaced CMFs in a growing primary cell wall by Frey-Wyssling (1954). In contrast, when physicists and engineers refer to models, they generally mean explicit equations that define quantities, their rates of change, their dependence on well-defined parameters, and so on. These two realms can come together in molecular dynamics (MD) models, where molecules, their properties, and their interactions are represented in silico by explicit quantities and equations. Validation of the model is essential and usually comes by way of comparison of model predictions with experimental results. Once validated, the model may be used to test various hypotheses about the structure–function relations of a material, to predict responses to novel conditions, and to gain molecular insights into material properties and processes which are often not available by other means.

### Length scale of cell wall models

Molecular models of cellulose and other wall components have been developed at quantum, atomistic, and coarse-grained (CG) levels, in order of increasing length scale. Quantum models have related the details of cellulose crystalline structure to results from vibrational and NMR spectroscopy and were used to estimate the energetics of different cross-sectional shapes of CMFs, for example, (Kubicki et al., 2018; Yang et al., 2018; Yang and Kubicki, 2020). In another vein, recent quantum modeling efforts concluded that dispersion forces (attractions due to induced dipoles) contributed substantially to the mechanical properties of cellulose, in some directions even more than did hydrogen bonding (Chen et al., 2021). This insight runs counter to the common notion that cellulose stiffness, insolubility, and other properties are primarily due to dense hydrogen bonding within the microfibril (Wohlert et al., 2021).

Moving to the next larger scale, atomistic modeling has been used to explore numerous cellulose properties. These include the mechanics of single microfibrils (Wu et al., 2013, 2014), force-dependent deformation and fracture (Chen et al., 2016; Molnar et al., 2018; Ciesielski et al., 2019), microfibril twisting (Zhao et al., 2013; Bu et al., 2015; Kannam et al., 2017) and surface properties (Mazeau, 2011; Trentin et al., 2021), to name just a few of the many studies in this prolific field. Atomistic modeling was also used to investigate the mechanism of binding between two microfibrils (Oehme et al., 2015), the binding between cellulose and matrix components including xyloglucan, xylan, and lignin (Zhang et al., 2011; Langan et al., 2014; Zhao et al., 2014; Falcoz-Vigne et al., 2017; Vermaas et al., 2019; Gupta et al., 2021) and the role of water in wall nanostructure (Cresswell et al., 2021). Atomistic models give many physical insights into the conformation and interactions of single and binary components of the cell wall, but larger, slower, and more complex systems or processes generally fall outside the capability of this method.

Next in scale is CG modeling which enables computational analysis of larger systems and slower processes beyond the reach of atomistic modeling (Zhang et al., 2009; Voth, 2017; Hafner et al., 2019; Shaebani et al., 2020). For this method, groups of atoms, for example, glucose residues, are represented by single particles called “beads” that are assigned appropriate physical properties, many in the form of mathematical equations, to mimic the properties of interest for the study. A recent review of CG models of cellulose (Mehandzhiyski and Zozoulenko, 2021) surveyed the use of this method to investigate cellulose structure, aggregation, and modifications by chemical and enzymatic treatments (Asztalos et al., 2012; Bellesia et al., 2012; López et al., 2015; Mehandzhiyski et al., 2020; Mehandzhiyski and Zozoulenko, 2021). As described in more detail in the next section, a CG model that included cellulose was recently developed to explore the molecular basis of the elasticity and plasticity of a primary cell wall (Zhang et al., 2021c).

Finally, numerous other mathematical approaches have been used to model selected aspects of cell wall mechanics and growth (reviewed in Geitmann and Ortega, 2009; Geitmann and Dyson, 2014; Smithers et al., 2019). They are often based on continuum mechanics or partial differential equations describing biochemical or biophysical processes, where individual polymers are not represented but their collective behaviors are abstracted to a higher level. Many recent plant biomechanics studies have adopted an engineering method—finite element modeling—to estimate stress patterns and deformation of plant cells or tissues (reviewed by Bidhendi and Geitmann, 2018). In these models, cell walls are commonly abstracted to thin shells represented as a mesh of elastic beams that transmit force to vertices with other beams, enabling computational simulations of changes in cell shape and wall stress upon changes in turgor pressure or applied force. The behaviors of individual cellulose, xyloglucan, and pectin chains are typically not represented in such models. An exception to this last statement is found in attempts to simulate aspects of wall elasticity with finite element models where polymers are represented as fixed elastic beams (Kha et al., 2010; Yi and Puri, 2012; Nili et al., 2015). Finite element models are well suited for assessing elastic deformations and stress patterns. To simulate growth, some additional steps are introduced. For instance, growth of pollen tubes and trichomes was simulated by a series of elastic stretches that were incrementally “fixed” by a renormalization procedure to remesh the wall and simulate addition of wall polymers (Fayant et al., 2010; Yanagisawa et al., 2015; Bidhendi and Geitmann, 2018). This mathematical procedure does not correspond to known mechanisms of growth and tacitly assumes that polymer motions underlying elastic and irreversible deformations are equivalent. However, AFM studies indicate otherwise (Zhang et al., 2017) and so too do the recent results of a CG model of cell wall mechanics, to be discussed in detail next. How differences between elastic and irreversible deformations might affect these growth simulations has not been assessed.

In comparison with finite element modeling, a larger set of realistic polymer properties and behaviors can be simulated by CG MD modeling, including polymer binding and unbinding as well as dynamic changes in polymer shapes, connectivity, and networking.

### A CG model of wall assembly and stretching mechanics

The CG model of Zhang et al. (2021c) explored several questions about cell wall assembly, structure, and mechanical function: can the noncovalent interactions and other physical properties of cell wall polymers account for their assembly into structures collectively resembling real cell walls? Which wall components resist in-plane tensile forces? What molecular motions contribute to the different phases of the wall’s stretching behavior? What are the molecular bases of wall elasticity, plasticity, and the yield threshold? In cross-lamellate cell walls, how are forces distributed in lamellae with cellulose oriented in different directions?

The model was based on the wall obtained by peeling the abaxial epidermis of onion scales. This polylamellate wall has been extensively studied by AFM (Zhang et al., 2014, 2016, 2017), various spectroscopic methods (Kafle et al., 2017; Huang et al., 2018; Ye et al., 2018, 2020) polysaccharide analysis (Wilson et al., 2021) and enzyme digestions combined with mechanical assays (Zhang et al., 2019; Wang et al., 2020), providing many details for building the model. A lamella is distinguished largely by its CMFs which are deposited in a common orientation, forming a reticulated network of CMF bundles (Ding et al., 2014; Zhang et al., 2014, 2016; Song et al., 2020; Figure 1). The surface lamella encompasses the entire face of the outer (periclinal) wall, but most of the lamellae in the periclinal wall do not extend into the side (anticlinal) walls which are much thinner and weaker than the thick outer periclinal wall. This pattern of cellulose deposition suggests that CSC movement around cell edges is severely limited. These inferences are consistent with the wall structure deduced for the epidermis of Arabidopsis hypocotyls (Crowell et al., 2011).

Note that this material differs from that used in many previous studies of onion epidermal peels obtained from the adaxial side of the onion scale, where the cells in the peel remain intact, alive, and capable of turgor pressure. The mechanics of these whole-cell epidermal strips have been studied for many years (Lintilhac et al., 2000; Wilson et al., 2000; Kerstens et al., 2001; Hepworth and Bruce, 2004; Suslov et al., 2009; Beauzamy et al., 2015; Bidhendi et al., 2020; Natonik-Białoń et al., 2020). They inevitably include a more complex cell geometry and mechanical contributions from turgor pressure and changes in protoplast volume.

Because CG modeling is a recent addition to the plant cell wall field, I provide some nontechnical details of the construction of the model and its usefulness in the following paragraphs.

#### Components of the CG wall 

**Figure 5:**
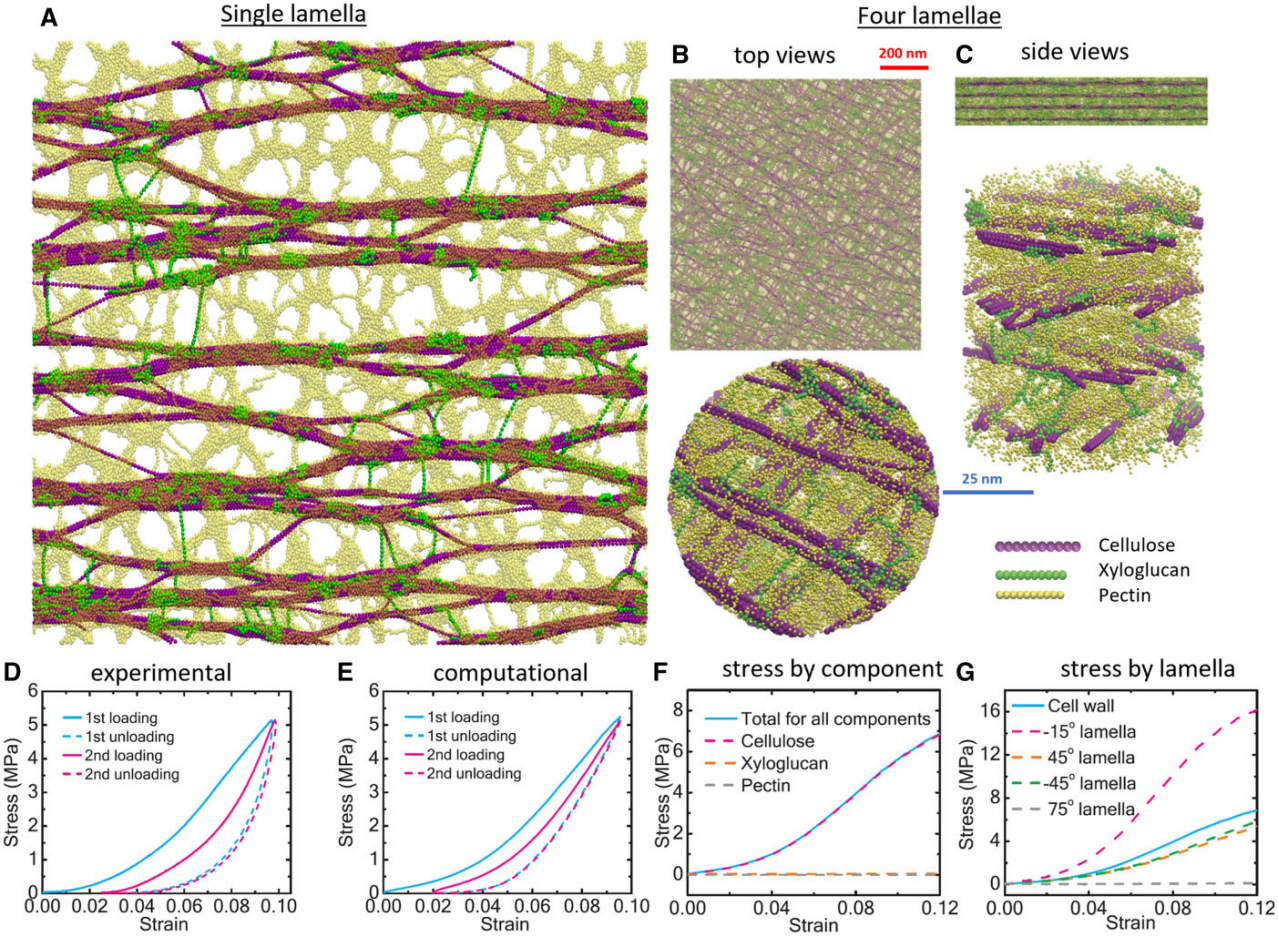
CG model of the structure and mechanics of the onion epidermal cell wall. A, Structure of a single lamella after equilibration. CMFs (purple) become bundled and form a cohesive network. Xyloglucans (green) bind to CMFs in different configurations: extended, coiled, trapped between CMFs in bundles, and tethers connecting CMFs. Pectin (yellow) forms a soft network with extensive but weak contacts to cellulose and xyloglucan. B, Tops views of a four-lamella CG wall. C, Side views of the same wall as in (B). The red 200-nm scale bar applies to the top images in (B) and (C) while the blue 25-mn scale bar applies to the lower images in (B) and (C). D–G, Different measures of cell wall mechanics (stress–strain curves). D, Experimental stress–strain curves of two cycles of extension by 10%, showing loading and unloading curves in both cycles. E, Computational simulation of the measurements in (D). F, Cellulose bears essentially all the stress during 12% extension. G, Stress is greatest in the lamellae where cellulose is oriented closest to the direction of stretching, the 15° lamella in this case. Adapted from Zhang et al. (2021c), with permission.

The model consisted of a square patch of cell wall, nearly 1 μm on a side, made of four lamellae with cellulose oriented in different directions (Figure 5). The real wall consists of many more lamellae, but four lamellae were deemed sufficient to capture the essential features of the cross-ply wall. The three most abundant polysaccharides (cellulose, xyloglucan, and HG) were represented as bead-and-spring models of polymers. They were parameterized in a top-down approach based on physical values from the literature. Each bead corresponded to a 3-nm length of the corresponding polymer, approximately six glycosyl units long. The springs between beads were assigned tensile and flexural stiffnesses corresponding to the Young’s modulus and persistence length for each of the polysaccharides. These parameters quantify the resistance of the polymers to tensile stretching and to bending, respectively. To simulate binding between polymers, the beads were assigned rules governing distance-dependent energies based on published experimental estimates of the binding strengths of polymer interactions. Cellulose–cellulose interactions were the strongest, followed by xyloglucan–cellulose and weaker interactions were assigned for the remaining pairwise interactions (pectin–cellulose, pectin–pectin, xyloglucan–xyloglucan, and xyloglucan–pectin). These CG representations approximate the physical properties considered relevant for polymer dynamics and interactions, but some structural features of these polymers are missing. For instance, the hydrophilic and hydrophobic surfaces of the cellulose crystal are not distinguished in this CG model; a supra-CG model of cellulose (Mehandzhiyski et al., 2020) could be used in future studies if this feature is deemed essential for a more sophisticated cell wall model. Likewise greater pectin complexity, such as methyl-esterified blocks, could be included, for example, see Pieczywek et al. (2020b), if warranted, as could variations in xyloglucan side chains. However, experimental data for CG parameterization of such polymer features is limited or nonexistent and sensitivity analyses suggest that these simulations would change in only minor ways upon inclusion of such features (see supplemental discussion of Zhang et al., 2021c).

#### Wall assembly

To construct a single lamella, cellulose was initially positioned in a common direction with some dispersion to mimic natural variations in cellulose alignment. HG and xyloglucan chains were randomly distributed in the remaining volume. Four stacked lamellae with different cellulose orientations were set up in this manner and the polymers were then allowed to interact to reach a low energy state. In the resulting structure (Figure 5), CMFs within each lamella assembled into a cohesive bundled network with morphological features resembling the actual wall. Xyloglucans were bound to cellulose surfaces, sometimes sandwiched between two microfibrils, sometimes in tether-like configurations, and sometimes in random coil shapes, resembling patterns detected with nanogold labeling of xyloglucan (Zheng et al., 2018). HG made an extensive, dynamic network displaying abundant contacts with cellulose surfaces. The model allowed movements of the polymer chains throughout the simulation box; nevertheless, the four cellulose networks remained distinct; xyloglucan and HG chains physically connected CMFs in adjacent lamellae. These results support the hypothesis that many of the nanoscale morphological features of the cell wall emerge from the collective physical interactions of these three wall polymers.

#### The molecular basis of wall mechanics

How do the mechanical properties of this emergent structure compare with those of the real cell wall? To address this question we stretched the wall model in silico and compared the resulting force/extension (stress/strain) curves with parallel experiments using real epidermal peels. The real walls were stretched in two cycles (Figure 5D). During the first stretch some of the deformation was irreversible due to wall plasticity. The second stretch was largely reversible (elastic). The unloading curves were identical in the two cycles and were notable for their very steep initial slope.

The CG simulation captured these complex stress/strain behaviors to a remarkable extent (Figure 5E). Moreover, inspection of the simulation results resolved questions about the molecular mechanics of the wall that have been difficult to address experimentally. In the simulation, tensile forces were transmitted primarily by the interconnected cellulose network, with little contribution from xyloglucan and HG (Figure 5F). This result is consistent with experiments showing that enzymatic digestion of these matrix polysaccharides had little effect on tensile stiffness of onion walls (Zhang et al., 2019). It is also consistent with the results of genetic removal of xyloglucans in Arabidopsis (Cavalier et al., 2008; Kim et al., 2020).

**Figure 6:**
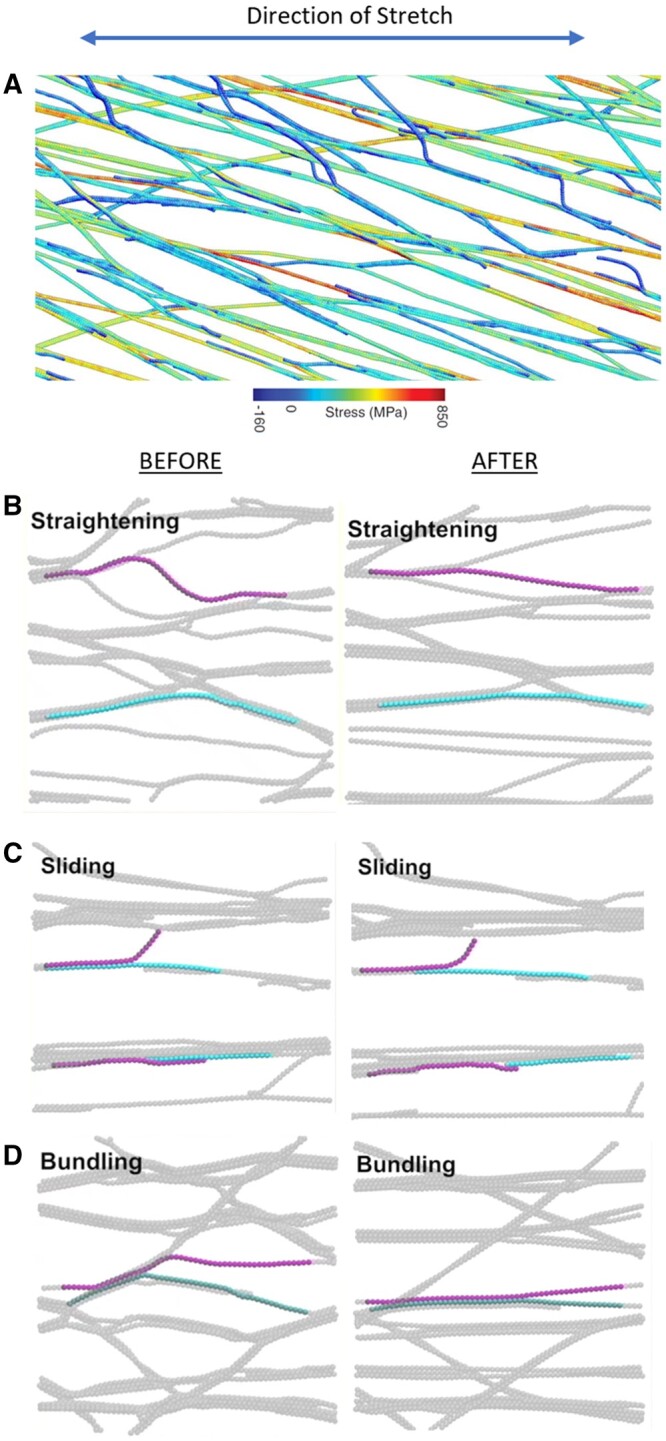
Examples of CMF dynamics during stretching of the CG cell wall. A, Tensile stress in CMFs is highly heterogeneous. Examples of two CMFs undergoing (B) straightening, (C) sliding and (D) changes in bundling. Adapted from Zhang et al. (2021c), with permission.

The four lamellae in the model contributed unequally to the wall’s stretch resistance, with the largest resistance coming from cellulose most closely aligned to the direction of stretch (Figure 5G). Close inspection of the stress within the cellulose network revealed a highly uneven stress distribution (Figure 6A) ranging from 850 MPa for some (but not all) of the CMFs aligned in the direction of stretch to −160 MPa for CMFs transverse to the direction of stretch. These transverse CMFs undergo compression and bending as the wall is stretched axially. These insights into the distributions of wall stress are important for attempts to understand how wall stresses may influence the orientation of microtubules, key players in cell morphogenesis (Williamson, 1990; Landrein and Hamant, 2013; Hamant et al., 2019; Trinh et al., 2021). This unevenness may factor into the nanoscale heterogeneity of stiffness seen in surface indentation of primary cell walls (Yakubov et al., 2016).

The stiffening of the wall seen at small strains (<5%) resulted from straightening of CMFs (Figure 6). At higher strains, the cellulose network began to yield as a result of sliding of cellulose in bundles. Such cellulose–cellulose sliding accounted for most of the plastic deformation in these stretching assays. The initial steepness of the unloading curve was explained as elastic unloading of the CMFs aligned in the stretch direction; the steepness is a reflection of the very high tensile stiffness (Young’s modulus) of CMFs. Cellulose bundling is observed to change reversibly during the second loading–unloading cycle of the CG wall; such changes in bundling dissipate mechanical energy and contribute to the hysteresis in this reversible cycle. Similar microfibril motions were observed experimentally by AFM during stretching experiments (Zhang et al., 2017).

#### Perspective

This CG model highlights the mechanical importance of direct cellulose interactions for transmission of tensile forces within the plane of the wall. In contrast, previous depictions of primary cell walls postulated well-spaced microfibrils, implying that the matrix transmits tensile forces between microfibrils. A well-spaced microfibril texture was featured in the early wall model of Frey-Wyssling (1954) and was carried forward in subsequent molecular-scale depictions (Keegstra et al., 1973; Albersheim, 1975; McCann and Roberts, 1991; Talbott and Ray, 1992b; Carpita, 1996; Somerville et al., 2004). These cartoons omit CMF–CMF junctions, which are important sites for force transmission in the CG model. Additionally, the molecular scale of these earlier depictions may be too small to capture important mechanical behavior of primary cell walls, where a scale of ∼500 nm or greater is needed because of the topology of microfibril bending and bundling.

Another important conclusion from the CG model is that cell wall plasticity arises from CMF sliding, somewhat resembling the action of an extension ladder, and that the plastic yield threshold depends on the strength of cellulose–cellulose contacts. This insight diverges from previous concepts where plastic deformation was thought to be controlled by the matrix (Nishitani and Masuda, 1981; Schopfer 2006). One (faulty) reason for considering the matrix to be the site of cell wall yielding was summarized by Haughton et al. (1968) who noted that the tensile modulus of cellulose is at least 100 times greater than that of growing walls; from this comparison they inferred that wall extensibility must be controlled by the softer matrix rather than cellulose. The flaw in this reasoning is brought out by the CG model which shows how the wall modulus (20–100 MPa) was determined primarily by straightening of CMFs (modulus 100× higher) (Zhang et al., 2021c). The load-bearing microfibrils comprised only ∼1%–2% of the relevant cross-section area of the wall, yet resisted nearly all of the mechanical force, with negligible direct contribution by the matrix. Nonetheless, the matrix may influence mechanical behaviors indirectly by interfering with CMF motions and formation of the CMF network. These considerations are important for calculating the shear forces involved in CMF sliding and in thinking about how cell wall stresses may be sensed by the cell to modulate microtubule orientations (Williamson, 1990; Trinh et al., 2021).

Despite its success in capturing wall plasticity, the CG model does not simulate cell wall creep. Plasticity is the time-independent irreversible deformation that occurs when the wall is stretched to a stress above the yield threshold whereas creep is the slow, time-dependent increase in wall length at constant stress. Creep is generally considered to be central to the mechanism of cell wall growth in surface area, while plastic deformation—a one-time response to an increase in wall stress—is not. Expansins can stimulate cell wall creep without increasing cell wall plasticity (Yuan et al., 2001; Zhang et al., 2019), and wall hydrolytic enzymes may increase wall plasticity without stimulating creep (Zhang et al., 2019; Wang et al., 2020). Thus the two processes are not tightly connected. These two forms of irreversible deformation likely share some molecular features, but further experimental and theoretical analyses are needed to clarify how they relate at the molecular level.

Finally, this CG study demonstrates a way to connect the physical properties of wall polymers with their assembly and resulting mechanics. The conclusions may be viewed as a set of proposals, hypotheses in need of further testing, that is, with other types of walls and with other approaches. The universe of plant cell walls is indeed large. There are even cases of CMFs that are well separated by a hydrated matrix, for example, in mistletoe (*Viscum album*) berries. They behave like a viscous mucilage, but when mechanically drawn into close alignment they form coherent, extensible fibers of high stiffness (Azuma et al., 2000; Horbelt et al., 2019).

## Mechanisms of irreversible wall enlargement

Wall enlargement in growing cells is directly coupled to cellular water uptake. This coupling hinges on stress relaxation of the cell wall, a process that reduces cell turgor (slightly) and thereby generates the water potential difference for cellular water uptake (Ray et al., 1972; Cosgrove, 1981a, 1993; Dumais, 2021). Within this conceptual framework, the molecular mechanism of wall stress relaxation is central to wall extensibility for cell growth.

Proposed mechanisms of cell wall stress relaxation and surface enlargement include: (1) physical yielding of the wall polymers to wall stresses by passive viscoelastic polymer creep; (2) active insertion of matrix materials, particularly HG, into the cell wall; and (3) protein-mediated chemorheological creep. These three potential mechanisms, briefly detailed below, are not mutually exclusive and it is theoretically possible that different mechanisms, or combinations thereof, operate in cell walls with different structures and at different time scales.

### Viscoelastic deformation

As a hydrated polymeric material, growing cell walls are often assumed to undergo viscoelastic extension, an idea consistent with the idea of the cell wall as a pectin hydrogel reinforced by CMFs (Oliveri et al., 2019). “Viscoelastic” generally means a material has a combination of elastic (reversible) and viscous (fluid-like) behaviors. However, there are difficulties with the view that wall viscoelasticity is the point of control of cell wall growth. First, isolated cell walls without active expansins behave like viscoelastic solids, which means their ability to extend without protein mediators of cell wall creep is very limited. When clamped in a stretching device and loaded to a constant force, primary walls without active expansins extend rapidly in the first seconds but the rate soon slows to a crawl, approaching a near-zero extension rate (<1% extension per hour) within a few minutes, for example, Probine and Preston (1962). In contrast, rapidly growing cell walls may extend in vivo at 10% per hour or more for many hours. Thus simple polymer viscoelasticity seems to contribute little to steady growth at constant turgor pressure. Second, wall extension rates in living tissues can be increased or decreased within seconds or minutes, yet measures of wall viscoelasticity do not change or the changes lag far behind the change in GR (Cleland, 1984; Cosgrove, 1988). In some cases changes in pectic viscosity run opposite to expectations from changes in GR (Nishitani and Masuda, 1981). Third, when lytic enzymes that depolymerize matrix polysaccharides are applied to cell walls under tension, the walls do not extend in response to matrix breakdown (Ruesink, 1969; Cosgrove and Durachko, 1994; Zhang et al., 2019), even though the matrix becomes demonstrably softer. Finally, changes in wall viscoelasticity are often poorly associated with changes in growth; see review by Taiz (1984). These behaviors are clarified by the CG model (above) where the wall lengthens by CMF–CMF sliding of the coherent cellulose network whereas the relatively soft matrix simply accommodates the cellulose movements. This might be different if the stiffness of the matrix matched or exceeded the stiffness of the cellulose network or CMF–CMF contacts were rare (Shedletzky et al., 1992).

### Insertion of HG

This idea is a form of the classical concept of “growth by intussusception,” which asserts that insertion of wall substances between wall material already present in the wall is the basis for its surface enlargement. Evidence cited in favor of this growth mechanism includes the rough association between wall synthesis and wall expansion in growing tissues, for example Ray (1962). On the other hand, wall synthesis is often not well coupled to wall extension (Bret-Harte et al., 1991; Bret-Harte and Talbott, 1993; Refregier et al., 2004; Ivakov et al., 2017). Moreover, isolated cell walls, when stretched at constant force to replace the physical effect of turgor, can creep for many hours without addition of wall substances (Cleland et al., 1987; Li et al., 1993). The idea of growth by intussusception has been revived recently in the form of various theories aiming to link insertion of HG to cell wall enlargement. (1) Rojas et al. (2011) proposed a continuum model for pollen tube enlargement that envisions a pectin-based wall held together by Ca^2+^ crosslinking of unesterified HG blocks. In the model, HG is secreted, de-esterified by PME, competes for Ca^2+^ at HG–Ca^2+^ junctions and thereby facilitates incorporation of new HG into the expanding wall. HG–Ca^2+^ junctions are thus hypothetical sites of wall stress relaxation induced chemically by unesterified HG in the wall. Evidence in favor of this proposal was summarized by Boyer (2016) for the giant-celled alga *Chara* and for pollen tube growth. The stability of the process critically depends on an adequate supply of Ca^2+^ to stabilize the dynamically changing HG junctions. (2) Ali and Traas (2016) proposed a related idea in which HG–Ca^2+^ junctions dynamically dissociate at a rate that increases with the tensile stress borne by pectin chains; these chains are depicted as composed of many HG chains linked linearly end-to-end by Ca^2+^ junctions. When HG junctions are forced apart by wall stress, unesterified HG chains are proposed to repair the breach by binding to the two formerly connected HG chains, forming new HG–Ca^2+^ junctions and elongating the pectin fiber. The authors called this “force-driven polymerization.” (3) Haas et al. (2020) offered a different concept of pectin intussusception to account for lobing of the anticlinal walls of Arabidopsis leaf pavement cells. They proposed that HG is deposited as packed crystalline nanofilaments between CMFs deposited in parallel order. Upon de-esterification by PME, the filaments are proposed to swell in diameter, increasing the lateral distance between CMFs. The authors described their model as a mechanism of wall enlargement independent of turgor-driven growth.

These three mechanisms emphasize pectins but the role of cellulose and stresses transmitted by cellulose is omitted. In models (a) and (b), HG junctions must have sufficient stability to withstand the high tensile forces generated by turgor if they are to be relevant to the wall stress relaxation process. It is not clear that this is the case as pectin gels are orders of magnitude softer than primary cell walls (Jarvis, 2002; Gawkowska et al., 2018; Williams, 2020). For model (c), some of its conclusions differ from other studies of lobing in pavement cells (Altartouri et al., 2019; Belteton et al., 2021; Liu et al., 2021), so further testing is called for (Cosgrove and Anderson, 2020; Chebli et al., 2021).

### Protein-mediated cell wall creep

In its earliest forms, this concept postulated the existence of wall-loosening enzymes that cut load-bearing matrix polymers, enabling the wall to yield irreversibly to turgor-generated cell wall stress in a sustained manner, that is, to undergo chemorheological creep. When I was a graduate student, this hypothesis was heavily favored by the cell wall community. Given the dominance of the tethered network model at that time, β-glucanases were frequently cited as potential candidates, but they never panned out. Xyloglucan endotransglycosylase (XET) was hypothesized to have this activity (Smith and Fry, 1991; Nishitani and Tominaga, 1992) and there is limited evidence for modification of wall mechanics by XET (Van Sandt et al., 2007), but the majority of the evidence on this question comes out against XET as a substantial wall-loosening enzyme (Cosgrove, 2016). In my view no plant enzyme has been documented to possess cell wall creep activity, even though many enzymes have at times been asserted to have such activity, for example, PME, pectinase, pectate lyase, xyloglucanase, cellulase, and others. Some bifunctional microbial endoglucanases (with xyloglucanase and cellulase activity) can induce plant cell wall creep (Park and Cosgrove, 2012b), but no plant equivalent has been discovered (Behar et al., 2021). The results of Zhang et al. (2019) showed that various wall lytic enzymes may soften cell walls (reduce their resistance to applied forces) yet not loosen the walls (not induce cell wall creep). The distinction between mechanical softening and ability to undergo sustained creep is important in considering potential wall-loosening enzymes.

In contrast to enzymes with wall lytic activity, no enzymatic activity has been detected for α-expansin proteins, yet they are potent facilitators of cell wall creep (McQueen-Mason et al., 1992; Cho and Kende, 1997; Cosgrove, 2000; Takahashi et al., 2006; Cosgrove, 2015, 2016). Their action is the basis of “acid growth,” originally associated with auxin action (Rayle and Cleland, 1992) but is generalizable to other developmental situations. At low pH (<5), α-expansins strongly stimulate cell wall creep, whereas their activity at neutral pH is reduced. Recent reports have linked pH-dependent changes in pectin physical chemistry to acid growth (Hocq et al., 2017; Arsuffi and Braybrook, 2018; Phyo et al., 2018), yet such changes alone are insufficient to induce acid growth. The relevant experiment supporting this conclusion was first published 30 years ago (McQueen-Mason et al., 1992) and has been replicated in numerous other tissues: inactivation of hypocotyl cell walls by brief heat or protease treatments eliminated their acid growth response which was restored by addition of purified α-expansin protein. Pectin physical chemistry clearly depends on pH, but the involvement of pectins in acid growth remains an open question in need of further investigation. Expansin activity is also promoted by thiol reducing reagents (Cosgrove, 1989; Li et al., 1993), which offers an unexplored pathway for cellular control of expansin action via redox control of the cell wall environment.

## Prospectus

When we initially characterized the action of α-expansins, we found that they weakened filter paper which we used as a bare-bones mimic of the complex plant cell wall (McQueen-Mason and Cosgrove, 1994). This phenomenon was later confirmed and extended with cellulose-based pellicles produced by *Acetobacter* (Whitney et al., 2000). Thus there was clear evidence from the start that α-expansins can loosen cellulose–cellulose junctions. We did not consider this action to represent α-expansin’s native mechanism of wall loosening because of the prevailing notion that the matrix fully coats microfibril surfaces, sterically hindering the formation of cellulose–cellulose contacts in native plant cell walls. I now think otherwise. More recent results delineated above indicate that cellulose–cellulose junctions are important determinants of wall mechanics, assembly and extensibility. Our understanding of these junctions and their origin is in its infancy. They may be of multiple kinds: CMF–CMF contacts may be direct, in some cases fusing crystallites together over short regions; a thin layer of water may separate CMF surfaces or a monolayer of xyloglucan may serve as a pliant adhesive between two CMFs. Disordered cellulose chains on the surface of two CMFs may intermingle with each other and with xyloglucan or other matrix polymers, forming an interface that resists low mechanical stress yet slides with higher stresses. New tools are needed to identify and characterize junctions between CMFs and to explore how growing plant cells may modulate their formation as a step in building their extensible cell walls.

## Notes

D.J.C. wrote the paper.

The author responsible for distribution of materials integral to the findings presented in this article in accordance with the policy described in the Instructions for Authors (https://academic.oup.com/plphys/pages/general-instructions) is Daniel Cosgrove (dcosgrove@psu.edu).
